# PubChem synonym filtering process using crowdsourcing

**DOI:** 10.1186/s13321-024-00868-3

**Published:** 2024-06-16

**Authors:** Sunghwan Kim, Bo Yu, Qingliang Li, Evan E. Bolton

**Affiliations:** National Center for Biotechnology Information, National Library of Medicine, National Institutes of Health, Bethesda, MD 20894 USA

**Keywords:** PubChem, Chemical database, Crowdsourcing, Crowdvoting, Chemical name-structure association, Medical Subject Headings (MeSH), Database search

## Abstract

PubChem (https://pubchem.ncbi.nlm.nih.gov) is a public chemical information resource containing more than 100 million unique chemical structures. One of the most requested tasks in PubChem and other chemical databases is to search chemicals by name (also commonly called a “chemical synonym”). PubChem performs this task by looking up chemical synonym-structure associations provided by individual depositors to PubChem. In addition, these synonyms are used for many purposes, including creating links between chemicals and PubMed articles (using Medical Subject Headings (MeSH) terms). However, these depositor-provided name-structure associations are subject to substantial discrepancies within and between depositors, making it difficult to unambiguously map a chemical name to a specific chemical structure. The present paper describes PubChem’s crowdsourcing-based synonym filtering strategy, which resolves inter- and intra-depositor discrepancies in synonym-structure associations as well as in the chemical-MeSH associations. The PubChem synonym filtering process was developed based on the analysis of four crowd-voting strategies, which differ in the consistency threshold value employed (60% vs 70%) and how to resolve intra-depositor discrepancies (a single vote vs. multiple votes per depositor) prior to inter-depositor crowd-voting. The agreement of voting was determined at six levels of chemical equivalency, which considers varying isotopic composition, stereochemistry, and connectivity of chemical structures and their primary components. While all four strategies showed comparable results, Strategy I (one vote per depositor with a 60% consistency threshold) resulted in the most synonyms assigned to a single chemical structure as well as the most synonym-structure associations disambiguated at the six chemical equivalency contexts. Based on the results of this study, Strategy I was implemented in PubChem’s filtering process that cleans up synonym-structure associations as well as chemical-MeSH associations. This consistency-based filtering process is designed to look for a consensus in name-structure associations but cannot attest to their correctness. As a result, it can fail to recognize correct name-structure associations (or incorrect ones), for example, when a synonym is provided by only one depositor or when many contributors are incorrect. However, this filtering process is an important starting point for quality control in name-structure associations in large chemical databases like PubChem.

**Supplementary Information:**

The online version contains supplementary material available at 10.1186/s13321-024-00868-3.

## Introduction

PubChem [1–3] is a public repository of information on chemical substances and their biological activities, developed and maintained by the U.S. National Institutes of Health. Since its launch in 2004, PubChem has grown rapidly and serves as a key chemical information resource for many research areas such as cheminformatics, chemical biology, and drug discovery. PubChem organizes its data into multiple data collections [4–6], including Substance, Compound, BioAssay, Protein, Gene, Pathway, Cell Line, Taxonomy, and Patent. Substance [4] archives chemical substance information provided by individual data contributors. Compound [4] stores unique chemical structures extracted from the Substance database. The descriptions and results of biological assays on chemical substances are contained in the BioAssay database [5]. The Protein, Gene, Pathway, Cell Line, and Taxonomy collections [6] contain chemical information specific to a given biological target (i.e., protein, gene, pathway, cell line, and taxon), along with annotations about the target, collected from curated and authoritative data sources. The Patent data collection provides chemicals mentioned in a patent, as well as the patent metadata (e.g., the title, abstract, inventor, assignee, and the priority/filing/grant/publication dates). Various aspects of PubChem, including data contents and organization, interfaces, programmatic access, and other relevant tools and services, are described in detail by our previous papers [1, 7–10].

One of the most common tasks requested by users of PubChem, as well as other chemical databases, is to search for chemical structures using a chemical name query. Performing this task requires the mapping of chemical names (also called “synonyms”) to chemical structures. In PubChem, the chemical name-structure associations are provided by individual data contributors. These associations are looked up when a PubChem user queries a chemical name to retrieve the corresponding chemical structure. In addition, these synonyms are used to generate associations between chemicals in PubChem and scientific articles in PubMed via Medical Subject Headings (MeSH) terms [11], where MeSH is a manually curated thesaurus used to index MEDLINE content within PubMed by the National Library of Medicine (NLM). MeSH consists of sets of terms naming descriptors in a hierarchical structure that permits searching at various levels of specificity, and many of these terms are chemical names. The primary terms in the MeSH vocabulary are called “Headings” or “Descriptors”. [There are also Supplementary Chemical Records (SCRs) that are mapped to one or more Descriptors and these are used by MeSH to index chemicals and drugs. See also: https://www.nlm.nih.gov/mesh/intro_record_types.html] Each MeSH Heading has a short description or definition, links to related headings, registry numbers (if applicable), and a list of synonyms or very similar terms (known as “entry terms”). When a PubChem chemical name matches a MeSH heading or one of its entry terms or registry numbers, an association is created between the MeSH heading and the chemical structure represented by the synonym. These chemical-MeSH associations are used in turn to computationally generate associations from a chemical in PubChem to PubMed articles linked with the MeSH Heading associated with that chemical [12]. The resulting chemical-publication associations allow users to quickly retrieve a list of publications that are related to a given chemical [12].

As pointed out in several studies [13–17], mapping chemical names to chemical structures is very error-prone, raising concerns over data quality in many public databases. Ideally, a chemical name should be as specific as possible, allowing one to identify its corresponding chemical structure without ambiguity. However, because depositor-provided synonym-structure associations stored in PubChem have considerable discrepancies within and between depositors, it is difficult to associate a chemical name to a specific chemical structure unambiguously. These discrepancies in the synonym-structure associations may be classified into two different types: (1) intra-depositor discrepancy and (2) inter-depositor discrepancy. Whereas the intra-depositor discrepancy occurs when a depositor assigns a single chemical name to different chemical structures, the inter-depositor discrepancy refers to the case in which different depositors use the same chemical name to represent different chemical structures. It should be noted that these discrepancies refer to the ambiguity of the association of a depositor-provided synonym with multiple chemical structures, as opposed to a chemical structure associated with multiple synonyms, because a chemical structure can have many names that specifically represent that structure (e.g., methyl alcohol and methanol refer to the same chemical structure).

Resolving the intra- and inter-depositor discrepancies in synonym-structure associations is an important part of data quality assurance efforts in PubChem. To achieve this, PubChem uses a synonym-structure association filtering process, which tries to assign each depositor-provided synonym to only one chemical structure, using a “crowdsourcing” approach. The term “crowdsourcing”, first coined by Howe [18], refers to the “outsourcing” of tasks to an “undefined public” (the crowd), rather than to a specific group of people. While the concept of crowdsourcing can date back from as early as the late seventeenth century [19], the global spread of the internet has made crowdsourcing increasingly common, with a well-known example being Wikipedia. The scientific community has also been employing this technique to tackle a wide range of problems [20–31]. Importantly, crowdsourcing has been suggested as a way to improve the quality of data in large databases [32–39].

In this paper, we present a basis for PubChem’s crowdsourcing-based synonym filtering strategy, which resolves inter- and intra-depositor discrepancies in synonym-structure associations as well as in the chemical-MeSH associations. Based on the results of this study, we discussed the synonym-filtering scheme currently implemented in PubChem since 2011.

## Methods

### Synonym data and pre-processing

The present study considered the synonym-structure associations for the substances that were successfully standardized through the PubChem structure standardization process [40]. This does not include substances with “auto-generated” structures. Because chemical structure information is not required for data submission to PubChem, some substances have no depositor-provided structures. For these substances, when the data contributor opts in, PubChem performs automated structure assignment based on depositor-provided synonyms, as explained in more detail elsewhere [40]. Those with auto-generated structures were excluded to avoid potential bias that may affect the consensus of synonym-structure associations among depositors. There were 10.3 million substances with auto-generated structures, which corresponded to 4.5% of all 229.5 million substances in PubChem (as of June 2017).

All synonyms for the substances considered in this study were downloaded from the PubChem Substance database (in June 2017). These synonyms were pre-processed, by changing all lower-case letters (a–z) to the upper-case letters (A–Z) and then converting curly brackets “{}” and square brackets “[]” into rounded brackets “()”. All MeSH headings, terms, and substance names were downloaded from the MeSH database [11], and pre-processed in the same way as the PubChem synonyms.

### Tracking of data associated with a synonym

Comparing synonym-structure associations between different substance depositors in PubChem involves two important issues. First, because of the absence of universal standards or rules for chemical structure representation, different PubChem depositors adopt different approaches based on their organizational needs, frequently leading to different representations for an “identical” chemical structure. PubChem addresses this issue through structure standardization [40], in which depositor-provided chemical structures in the Substance data collection are validated and normalized, and unique standardized structures are extracted and stored in the Compound data collection. Records in the Substance and Compound collections are assigned to numeric identifiers, called substance identifier (SID) and compound identifier (CID), respectively. It should be emphasized that, because there are no general rules for structure standardization, the uniqueness of chemical structures in the PubChem Compound collection is very subjective. More detailed information on PubChem structure standardization is described elsewhere [40].

The second issue is the perception of the “sameness” of chemical structures. For example, different depositors have different views on how to treat stereochemistry, isotopism, and tautomerism when determining whether two chemicals are the same as each other. To address this perception of”sameness”, PubChem allows users to find identical molecules in the following contexts:

- same, connectivity: the molecules that have the same chemical connectivity, ignoring isotopes and stereochemistry.
- same, stereo: the molecules that have the same connectivity *and* stereochemistry, but ignoring isotopes.
- same, isotope: the molecules that have the same connectivity *and* isotopes, but ignoring stereochemistry.
- same, any tautomer: the molecules are tautomers of each other (when ignoring isotope and stereochemistry), especially when considering the presence of heating, solvents, and/or a catalytic amount of acid or base.

**Fig. 1:**
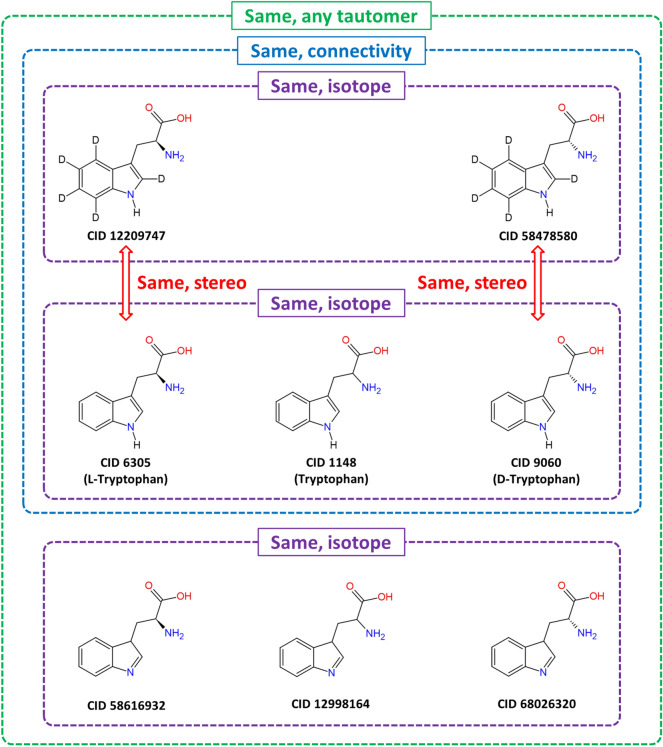
Different contexts of the “sameness” of chemical structures in PubChem. Tryptophan (CID 1148) and the other seven CIDs are tautomers of each other when isotope and stereochemistry are ignored (the “same, any tautomer” level). They are divided into two groups at the “same, connectivity” level. The group of five CIDs are further broken down at the “same isotope” and “same stereo” levels. See text for the definition of the four contexts of the “sameness”

These different contexts of the chemical equivalency of PubChem Compound records are illustrated in Fig. 1, with tryptophan as an example. Note that, if chemical structures in the PubChem Substance database have the same connectivity, stereochemistry, and isotopes after standardization, they are assigned to a common identifier (i.e., to the same CID) in the PubChem Compound database, and the association between their SIDs and this CID is generated. As a result, any two CIDs in the PubChem Compound database cannot simultaneously have the same connectivity, stereochemistry, and isotope. It is also noteworthy that PubChem merges different tautomeric forms of a given chemical into a single representative form through the chemical structure standardization process [40]. However, whereas this process works well for most chemical structures in PubChem, there are some edge cases, in which different tautomers are standardized into different forms, as exemplified in Fig. 1.

The ambiguity of synonym-structure associations may also arise from how to deal with mixtures or salt forms of a molecule. For example, although the drug name “Lipitor” is typically used to refer to the active ingredient “atorvastatin calcium”, it is also often used to indicate “atorvastatin” because the atorvastatin moiety of atorvastatin calcium is the primary ingredient attributed to the pharmacological effects of the drug. To help resolve such ambiguities, when a molecule has one or more covalent units, PubChem determines a parent component of the molecule, which is conceptually the important part of the molecule. Specifically, a parent compound must have at least one carbon atom and contain at least 70% of the heavy (non-hydrogen) atoms of all the unique covalent units (ignoring stoichiometry).

In the present study, the synonym–SID pairs were generated only for synonyms from SIDs with associated CIDs. For each of the synonyms, its associated depositor ID and SID were stored. In addition, standardized chemical structures in the PubChem Compound database were also tracked at six different levels of chemical structure equivalency (Table 1), using different “flavors” of CACTVS hash codes [41, 42], which are computed for each standardized structure and used for the final mapping from substance records to entries in the Compound database at the end of the structure standardization process [40].

**Table 1:** Six flavors of chemical structure information used to determine chemical equivalency

Abbreviation	CACTVS hash code used	Description
CID	CID hash code	In addition to atom connectivity, both isotopism and stereochemistry are considered to determine chemical structure equivalency. In practice, this category can be further classified into two categories: (1) CID-STD, in which indicates unanimity in synonym-structure association (meaning that the structure standardization alone can disambiguate synonym-structure association) and (2) CID-FILT, in which a consensus in synonym-structure association is reached at a level less than 100% (meaning that both structure standardization and synonym filtering are necessary to disambiguate synonym-structure association
STE	CID stereo hash code	In addition to atom connectivity, stereochemistry is considered to determine chemical structure equivalency. Information on isotopes is ignored
PCID	Parent CID hash code	Applicable only to multicomponent compounds. Same as CID, except that the parent compound’s hash code is used
PSTE	Parent CID stereo hash code	Applicable only to multicomponent compounds. Same as STE, except that the parent compound’s stereo hash code is used
CON	CID connectivity hash code	Only atom connectivity is considered for chemical structure equivalency. Neither stereochemistry nor isotopism is considered
PCON	Parent CID connectivity hash code	Applicable only to multicomponent compounds. Same as CON, except that the parent compound’s connectivity hash code is used

The different degrees of “sameness” used in this study were designed to preserve (to the extent possible) the stereospecificity of a chemical name. If structural consistency was not found at a given level, the next most specific level of “sameness” was used, where the order of specificity was (with the first being most specific and the last being the least specific): CID > STE > PCID > PSTE > CON > PCON

### Four crowd-voting schemes for resolving synonym-structure discrepancies

Synonym-structure discrepancies may be resolved using a crowd-voting strategy that looks for a consensus among different PubChem depositors on what chemical structure a given synonym refers to. In designing such a voting strategy, it is necessary to consider how to deal with intra-depositor discrepancies that may exist even within data from the same depositor. The two simplest ways to address this issue are the “one vote per depositor” and “many votes per depositor” approaches. In the “one vote per depositor” approach, an intra-depositor voting, which looks for a consensus within the depositor on the synonym-structure association, is performed for each depositor, and then only one structure per depositor determined from the intra-depositor voting is used for a subsequent inter-depositor crowd-voting. On the other hand, in the “many votes per depositor” approach, the intra-depositor discrepancies are ignored and all chemical structures from each depositor are used for the inter-depositor crowd-voting.

In both the intra- and inter-depositor voting schemes, an agreement was reached when more than a certain percentage of all chemical structures associated with a given synonym are the same structures. This strategy raised two important questions: what “percentage threshold” should be used and what the meaning of the “same” structures should be. While the choice of a threshold for agreement is inevitably arbitrary, two different thresholds (60% and 70%) were tested. [As a side note, the choice of 60% and 70% thresholds reflect a major consideration that most chemical names have relatively few data contributors such that most chemical names have very few cases that can pass a larger than 50% majority threshold when the vote is not unanimous, e.g., 2-out-of-3, 3-out-of-4, 3-out-of-5, 4-out-of-5, etc.] As a result, four voting scenarios, designated as Strategies I through IV, were tested in the present study, as summarized in Table 2. In addition, the sameness of chemical structures was determined at six different levels, using the six levels of compound hash codes of the standardized structures in PubChem associated with a given synonym (as shown in Table 1). The initial step of the voting considered all CID hash codes of the structures associated with the synonym to check whether the percentage of a certain CID hash code exceeds the threshold for agreement. If such a CID existed, it was considered that there was an agreement that the synonym best represents the chemical structure represented by that CID hash code. If no CID exceeds the threshold, it was considered that no agreement was reached at this “sameness” level, and then another voting at a different “sameness” level was performed using all CID stereo (STE) hash codes associated with the synonym. If no agreement was found, the voting was then further repeated at the other levels of “sameness” [i.e., in the order of same parent CID (PCID) → parent CID stereo (PSTE) → CID connectivity (CON) → parent CID connectivity (PCON)], until an agreement was reached (see Table 1). If no agreement is found, then no CIDs are associated to the given synonym chemical name.

**Table 2:** Four different crowd-voting strategies tested in the present study

Strategy	Number of votes per depositor	Consistency threshold (%)
I	Single	60
II	Single	70
III	Multiple	60
IV	Multiple	70

### MeSH filtering

In this study, each MeSH heading is assigned to an integer identifier, called MNID (which means “MeSH numeric ID”). It is a numeric representation of a MeSH unique ID (which begins with a letter). For example, MNIDs 68001241 and 2009860 (for aspirin and sildenafil citrate) are equivalent to MeSH unique IDs “D001241” and “D000068677”, respectively. The use of MNIDs (rather than MeSH unique IDs) speeds up database queries, especially when joining multiple tables. They originate from and are used by the NCBI MeSH Entrez interface (https://www.ncbi.nlm.nih.gov/mesh).

The associations between PubChem compound records (represented by CID) and MeSH records (represented by MNID) were created by matching the filtered synonyms from each of the four filtering strategies (strategies I through IV in Table 2) with MeSH headings and their entry terms and registry numbers. This often resulted in a compound being associated with many MeSH records, although it was desired to provide PubChem users with the most relevant MeSH heading for a given chemical. Therefore, the CID-MNID associations were filtered further using crowd-voting with a consensus threshold of 50%. This MeSH filtering effectively prevents a compound from being associated with more than two MeSH records.

## Results

### Statistics of unique synonyms and synonym-SID pairs

**Fig. 2:**
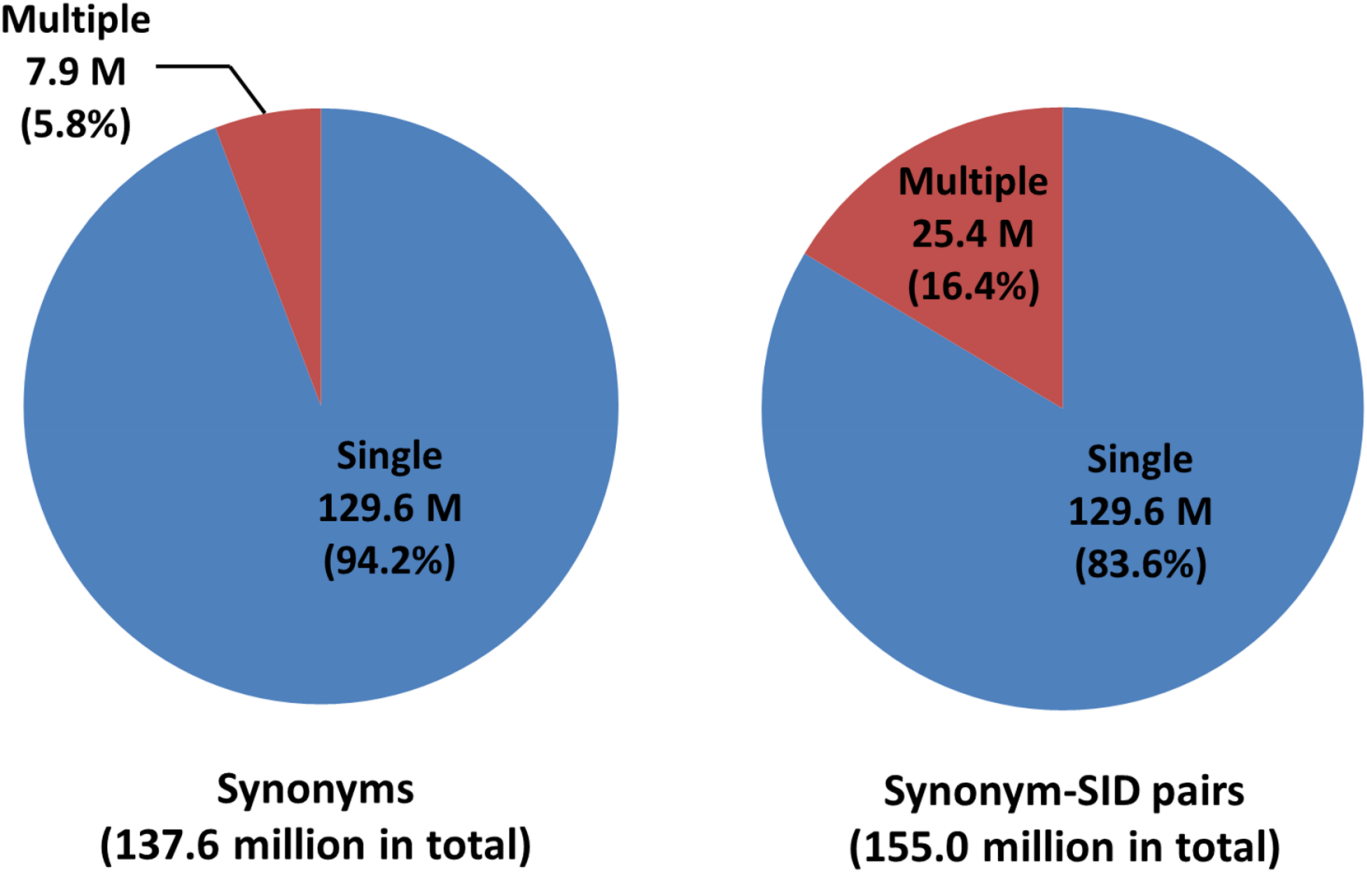
Number of unique depositor-provided chemical synonyms (left) and synonym-SID pairs (right). In the left panel, “single” (blue) and “multiple” (red) indicate unique chemical synonyms that occur only once and multiple times, respectively. In the right panel, “single” and “multiple” represent the synonym-SID pairs that involve the unique synonyms occurring once and multiple times, respectively

The Substance database had 229.5 million substance records at the time of initial paper writing (in June 2017). Among them, 220.5 million substances were successfully standardized through the PubChem structure standardization process [40], leading to 88.9 million unique structures in the Compound database. The depositor-supplied synonyms for the 220.5 million SIDs that had associated CIDs were downloaded and pre-processed as described in the “Methods” section, resulting in 137.6 million unique synonyms and 155 million synonym-SID pairs (see Fig. 2). About 94% of these synonyms (129.6 million synonyms) occurred only once in the Substance database and it is reasonable to map these synonyms to the structures represented by their associated SIDs. On the other hand, 7.9 million synonyms (5.8% of all unique synonyms) occurred multiple times, and are associated with 16.9 million SIDs, giving rise to 25.4 million synonym-SID pairs (16.4% of all synonym-SID pairs). These synonyms are potentially subject to intra- and/or inter-depositor inconsistencies in the name-to-structure mapping, which the synonym cleaning process described in this paper aims to address.

It is worth mentioning that the majority of synonyms that occur only once are various types of identifiers used by data sources, such as product identifiers from chemical vendors’ catalogs, record identifiers from scientific databases, sample identifiers from high-throughput screening facilities, and the like. The association of these identifiers with chemical structures is defined by the individual data sources. Strictly speaking, these identifiers are not chemical names, although the data depositors submit them as chemical synonyms. Another type of synonym typically occurring only once in PubChem is systematic chemical names generated from chemical structures using a computer program. While little ambiguity exists in what structures these systematic names mean, they are often long (and therefore less likely to be used for user queries and shared/reused as synonyms). For example, the systematic IUPAC name for “lipitor” (atorvastatin calcium; CID 60822) is “calcium; (3*R*,5*R*)-7-[2-(4-fluorophenyl)-3-phenyl-4-(phenylcarbamoyl)-5-propan-2-ylpyrrol-1-yl]-3,5-dihydroxyheptanoate”, which is 116 characters long. Short(er) names are often preferred (by humans and machines) and chemicals that have lots of information are often assigned short names, such as “lipitor” in the example above. Indeed, the most common chemical name queries are common names (e.g., “glucose”) or brand names (e.g., “lipitor”). These types of names are also frequently used by many PubChem depositors and hence subject to greater variability and ambiguity in their association with chemical structures, which is addressed (in part) by our crowdsourcing-based synonym filtering process.

**Fig. 3:**
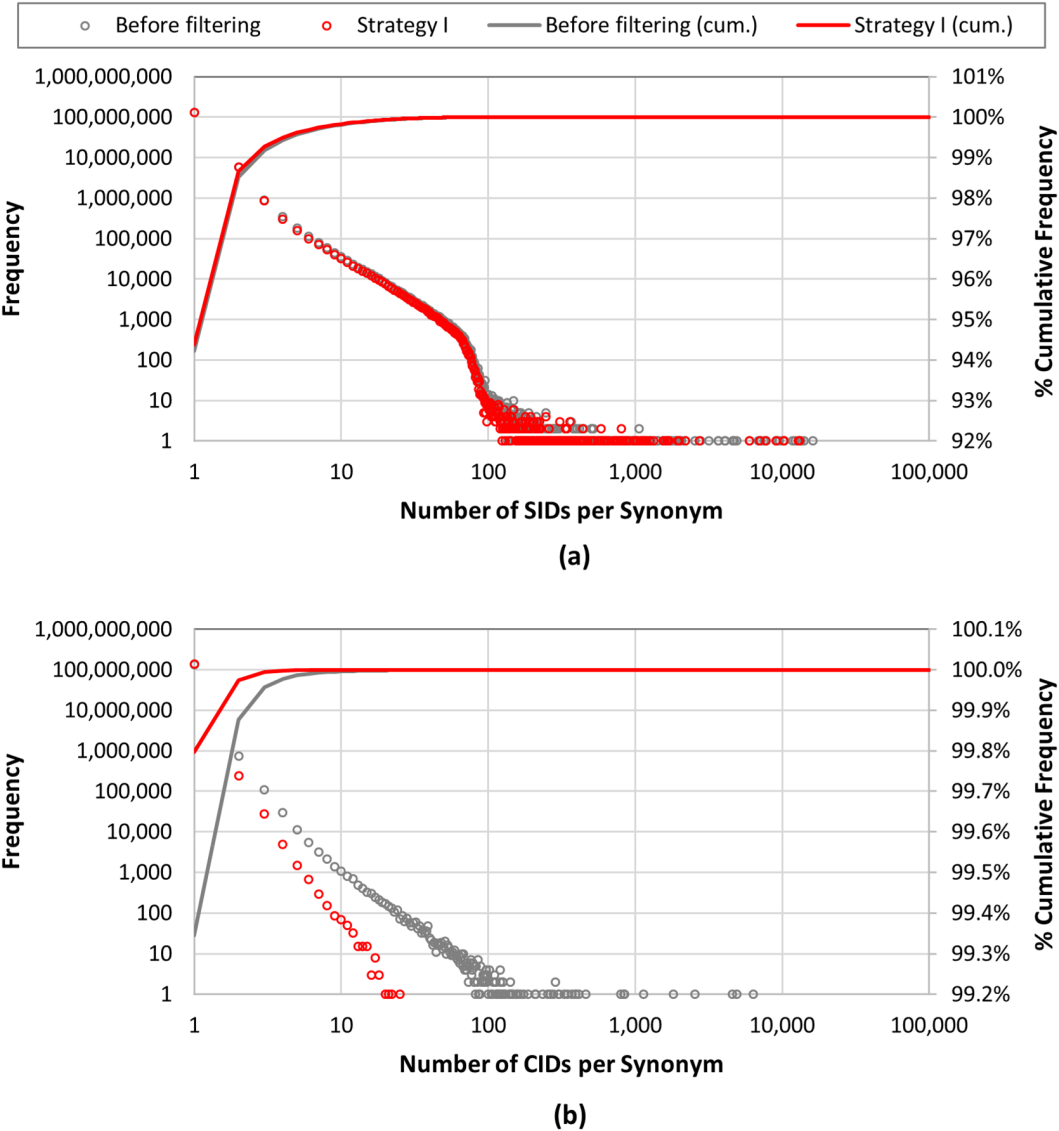
Distribution of the per-synonym SID and CID counts before and after synonym filtering using Strategy I: (**a**) the number of SIDs per synonym and (**b**) the number of CIDs per synonym. [The distributions for the other three filtering strategies considered in the present study are provided as a supplementary material (Additional file 1).]

In Fig. 3, the distribution of the per-synonym SID and CID counts before synonym filtering are compared with the values after filtering using Strategy I. Because the after-filtering distributions from Strategies II, III, and IV were very similar to those from Strategy I, they are not shown here but provided as a supplementary material (Additional file 1). Whereas most synonyms were associated with only a few SIDs before filtering, some appeared for more than 10,000 SIDs. It is noteworthy that the Substance database contains redundant structures submitted by individual data contributors and that PubChem’s standardization process takes care of this redundancy by extracting the unique chemical structures from the Substance database and storing them in the Compound database. Therefore, the CID count per synonym is expected to be smaller than the SID count per synonym, as shown in Fig. 3. However, many synonyms before filtering are still associated with as many as hundreds of or even thousands of CIDs.

**Fig. 4:**
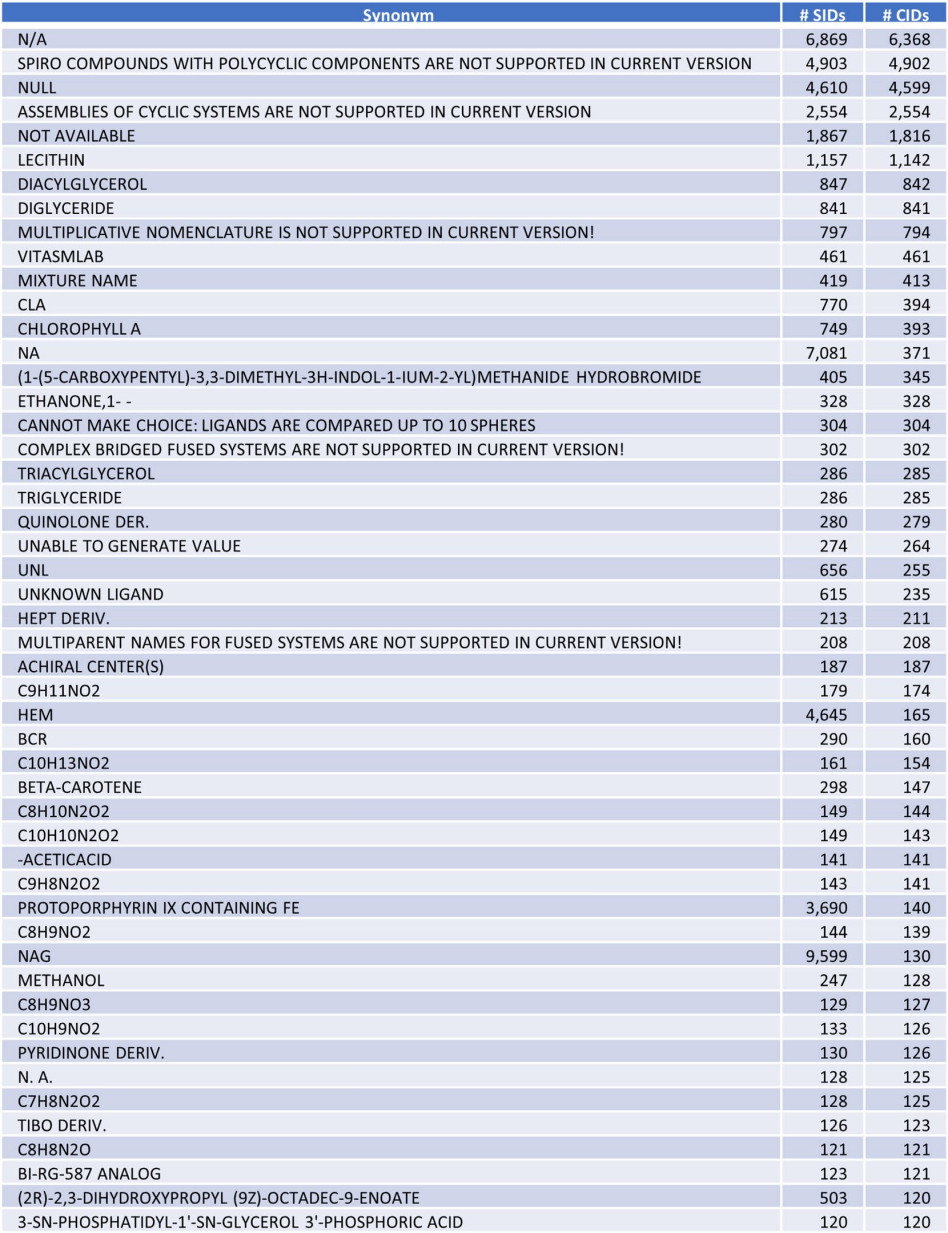
Top-50 depositor-provided chemical synonyms associated with the largest number of unique structures before synonym filtering, along with the number of associated PubChem SID and PubChem CID records

Figure 4 lists the top-50 depositor-provided synonyms associated with the largest number of CIDs before synonym filtering, along with the number of associated SIDs and CIDs, exemplifying common issues concerning depositor-provided synonyms. Many synonyms in PubChem are not chemical names, but molecular formulas (e.g., “C9H11NO2”) or abbreviations (e.g., “NAG” for “*N*-Acetyl-d-Glucosamine”; “CLA” for “Clarithromycin” or “Chlorophyll *A*”). [It is noteworthy that these abbreviations are also used as chemical identifiers in other scientific databases: for example, both “NAG” and “CLA” are used as ligand codes in the Protein Data Bank.] Sometimes, depositor-provided synonyms end with a word like “analog”, “analogue”, or “derivative” (often abbreviated as “deriv.”, or “der.”). Such synonyms are more like descriptions, rather than chemical names. With that said, the use of a short description as a chemical name (e.g., “PROTOPORPHYRIN IX CONTAINING FE” (140 CIDs)) is also common.

Interestingly, a group of the most commonly occurring synonyms before filtering includes “NULL” (4,610 SIDs), “Not Available” (1,867 SIDs) and its abbreviations such as “N/A” (6,869 SIDs) and “N.A.” (128 SIDs) (see Fig. 4). Another abbreviation for “Not Available” is “NA” (7,081 SIDs), which is also used as the atomic symbol of sodium by some data contributors. As a result, the chemical structures associated with “NA” include sodium atom/ion as well as other structures whose names are indicated as “NA” by data depositors. Another interesting aspect is that some synonyms were presumably error messages thrown out while chemical names were generated using a computer program. Because data sources often deal with millions of chemicals, it is not feasible to manually curate their names. Yet, it should be relatively easy for data sources to check if many records are associated to the same chemical name. As a best practice, users of chemical name generation software should be aware of and check for the chemical name generation failure output.

As exemplified in Fig. 4, depositors may provide virtually anything as chemical names, including molecular formulas, abbreviations, general descriptions, and error messages. Ideally, the PubChem synonym filtering process should be able to detect and remove these non-chemical names from depositor-provided synonyms. However, it also sheds light on the varied use cases for so-called “chemical names” across chemistry that blur the lines between a chemical name and a chemical description, with annotation and metadata often included in the context of a chemical synonym.

### Crowd-voting for resolving synonym-structure discrepancy

Table 3 summarizes the results of the synonym filtering through four crowd-voting strategies. While Strategies I and III filtered out ~ 230 thousand synonyms, Strategies II and IV filtered out ~ 300 thousand synonyms. This is due to Strategies II and IV using a higher consensus threshold (70%) than the other two strategies (60%). When Strategies II and IV are employed, it is much more difficult to reach a consensus on the synonym-structure correspondence, increasing the number of synonyms filtered out. For the same reason, Strategies II and IV resulted in fewer synonyms associated with a single CID than Strategies I and III. One could argue that any consensus threshold > 50% should be sufficient; however, PubChem aspires for more than a simple majority to form a consensus. Yet, a super majority is difficult to achieve in practice given the relatively infrequent use of the same chemical name by multiple data contributors. Thus, when considering this case of relatively few (e.g., between three and five) data contributors of the same chemical name, a minimum of 2-out-of-3, 3-out-of-4, and 3-out-of-5 are considered as being sufficient for there to be a consensus between data contributors at a 60% threshold; whereas a 70% threshold would require 3-out-of-3, 3-out-of-4, and 4-out-of-5 for there to be a consensus.

**Table 3:** Synonym counts before and after synonym filtering using four different strategies

	Before filtering	After filtering			
		Strategy I	Strategy II	Strategy III	Strategy IV
Synonym counts before and after filtering					
Synonyms before filtering	137,555,572	–	–	–	–
Synonyms filtered out	–	226,814	300,697	234,216	313,773
Synonyms remained	–	137,328,758	137,254,875	137,321,356	137,241,799
Synonyms with a single SID (A)	129,609,358	129,613,056	129,613,155	129,609,358	129,609,358
Synonyms with multiple SIDs (B = C + D)	7,946,214	7,715,702	7,641,720	7,711,998	7,632,441
- Assigned to a single CID (C)	7,045,461	7,437,677	7,288,184	7,429,005	7,268,388
- Assigned to multiple CIDs (D)	900,753	278,025	353,536	282,993	364,053
Synonyms with a single CID (E = A + C)	136,654,819	137,050,733	136,901,339	137,038,363	136,877,746
Synonym count differences between before- and after-filtering					
Synonyms with a single SID (ΔA)		3698	3797	0	0
Synonyms with multiple SIDs (ΔB = ΔC + ΔD)		− 230,512	− 304,494	− 234,216	− 313,773
- Assigned to a single CID (ΔC)		392,216	242,723	383,544	222,927
- Assigned to multiple CIDs (ΔD)		− 622,728	− 547,217	− 617,760	− 536,700
Synonyms with a single CID (ΔE = ΔA + ΔC)		395,914	246,520	383,544	222,927

The differences in synonym counts are computed by subtracting the pre-filtering values from the post-filtering values

On the other hand, whether to resolve intra-depositor discrepancies (i.e., single vote *vs.* multiple votes) made a relatively small impact on the filtering results, compared to the effects of the consensus threshold, although the multiple-votes approaches (Strategies III and IV) filtered out more synonyms. A notable difference between the single-vote and multiple-votes approaches was that the single-vote approach increased the number of synonyms with a single SID, whereas such an increase was not observed from the multiple-vote approach. This happens when one depositor associates a synonym with a single SID and another depositor associates that synonym with multiple SIDs (for simplicity, suppose that they are depositors A and B, respectively). For depositor B, the intra-depositor consensus cannot be reached, and all its synonym-SID associations are ignored in the subsequent inter-depositor voting stage. As a result, only the synonym-SID association from depositor A is considered in the inter-depositor voting and included in the filtered synonym list.

Before synonym filtering, 129.6 million synonyms (94.2%) were associated with only one SID, and therefore one CID (Table 3). These synonyms are mapped to the structures represented by their SIDs after structure standardization (i.e., the CIDs). These synonyms are not appropriate to process through the crowdvoting-based synonym filtering approach, which looks for a consensus among multiple name-structure associations. However, the majority of the synonyms appearing only once are identifiers used in various chemical databases and vendor catalogs and machine-generated systematic IUPAC names.

Among those associated with multiple SIDs, an additional seven million synonyms are associated with a single CID, meaning that the synonym unanimously represents the same standardized structure. When these synonyms are fed to the synonym filtering process, they reach a consensus at the 100% threshold (because there is only one CID for these synonyms). These cases are special cases in synonym filtering because the synonym-structure association is disambiguated through structure standardization alone prior to crowd-voting. With that said, the synonyms whose meanings were disambiguated at the CID level of chemical equivalency can be further classified into two groups:

- Those disambiguated with a name-structure association consensus of 100% (i.e., those disambiguated through structure standardization alone) (denoted as “CID-STD”).
- Those disambiguated with a consensus of less than 100% (denoted as “CID-FILT”).

**Fig. 5:**
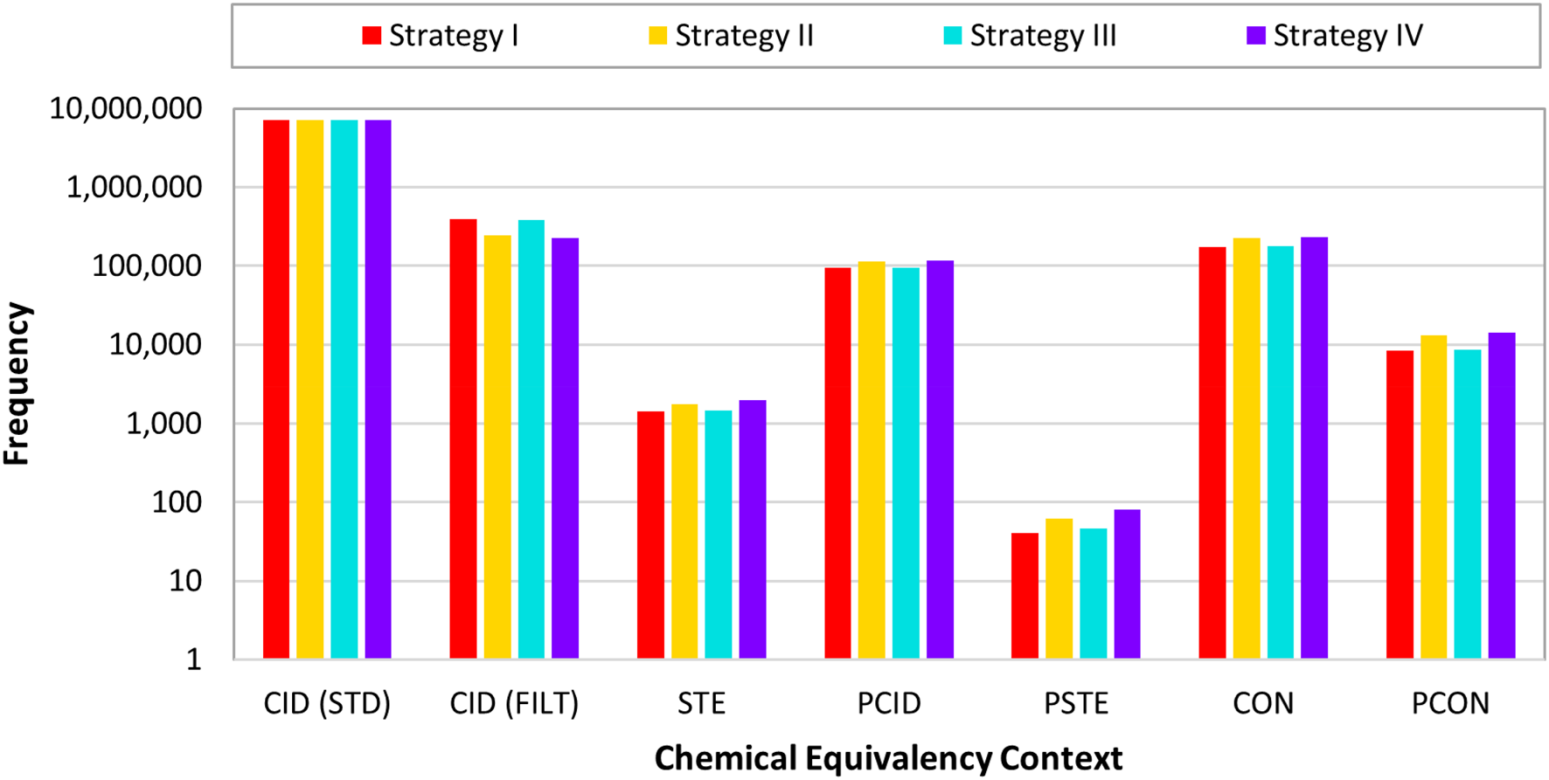
Synonym counts disambiguated for different chemical equivalency contexts considered during the synonym filtering using four strategies. See Tables 1 and 2 for the definition of the six chemical equivalence contexts and the four synonym filtering strategies. The largest number of synonyms are disambiguated at the CID level, while the least number of synonyms are disambiguated at the STE and PSTE levels

Table 4 and Figure 5 compare the number of synonyms resolved at each level of chemical equivalency (Table 1). Most synonyms were disambiguated at the CID level (corresponding to the CID-STD and CID-FIL combined). Especially, the fact that the largest number of synonyms are resolved at the CID-STD level indicates the importance of structure standardization upon the cleaning of name-structure associations. The smallest number of synonyms are resolved at the STE and PSTE levels, indicating that stereochemistry is a common issue in synonym-structure mapping (especially when considering the count of consistency cases resolved at the CON and PCON levels, where variability in stereochemistry is allowed, among other aspects).

**Table 4:** Number of synonyms that were successfully assigned to chemical structures as a function of level of chemical equivalency context during the synonym filtering, where the consistency order of CID > STE > PCID > PSTE > CON > PCON was used (CID being the most specific and PCON the least specific) and where consistency is reported only at the first most specific level of chemical equivalence

Context	Strategy I	Strategy II	Strategy III	Strategy IV
CID	7,441,375	7,291,981	7,428,814	7,268,223
- Standardization	7,045,461	7,045,461	7,045,461	7,045,461
- Synonym filtering	395,914	246,520	383,544	222,927
STE	1439	1741	1478	1948
PCID	93,566	113,122	95,301	116,681
PSTE	41	62	46	80
CON	174,717	225,459	177,679	231,353
PCON	8262	13,152	8680	14,156
Total	7,719,400	7,645,517	7,711,998	7,632,441

See the text and Table 1 for the description of the three-letter abbreviations, which represent different chemical equivalency contexts

**Fig. 6:**
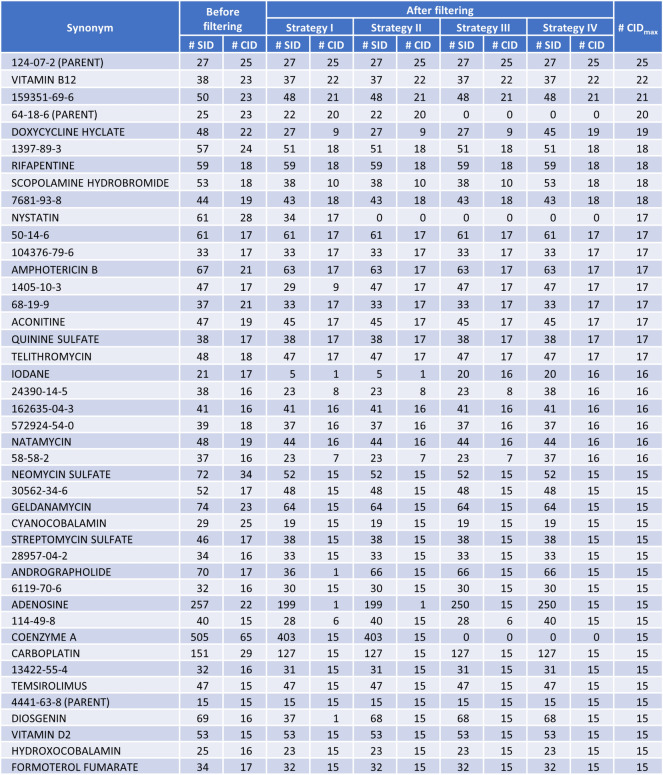
Synonyms associated with 15 CIDs or more after synonym filtering using any of the four different strategies, along with the number of SIDs and CIDs before and after filtering. The synonyms are sorted by the largest value among the after-filtering CID counts for the four filtering strategies

Figure 6 lists synonyms associated with 15 CIDs or more after synonym filtering using any of the four strategies. For all four strategies, the synonym with the most CIDs after filtering was “124-07-2 (Parent)”, associated with 25 CIDs after all filtering. These CIDs are various salt forms of octanoic acid (CID 379), whose CAS registry number is “124-07-2”. It seems to be a common practice that mixtures or salts are designated with the synonym of their major component. The synonym with the second most CIDs was “Vitamin B12”, which was associated with 22 CIDs. Some of these CIDs are mixtures containing Vitamin B12, while others correspond to structures with the same connectivity and different stereo specifications.

### Crowd-voting for MNID-CID mapping

In PubChem, MNID-CID mapping is done through two steps. First, filtered synonyms are matched with MeSH headings, entry terms, and registry numbers. If a match is found, an association is created between the MNID and the CID associated with the matched synonyms. In the second step, the generated MNID-CID associations are passed through MeSH filtering, which restricts a given CID to have no more than two MNIDs.

The impact of synonym filtering and MeSH filtering upon CID-MNID associations are summarized in Table 5. As shown in Table 5, when unfiltered synonyms were used, matching between unfiltered synonyms and MeSH terms resulted in 89,086 MeSH records being associated with any CID. Among them, 38,751 MeSH MNIDs (43%) were associated with multiple CIDs. Synonym filtering (without MeSH filtering) reduces this number to ~ 20 thousand, depending on the filtering strategies employed. It indicates that the MNID-CID associations became more specific upon synonym filtering.

**Table 5:** Statistics for MNID-CID mapping through synonym filtering and MeSH filtering using four different approaches

	Unfiltered	Strategy I	Strategy II	Strategy III	Strategy IV
Without MeSH filtering					
MNIDs with CID(s)	89,086	87,548	86,510	87,450	86,355
MNIDs with a single CID	50,335	67,029	65,348	66,781	65,021
MNIDs with multiple CIDs	38,751	20,519	21,162	20,669	21,334
CIDs with MNIDs	160,184	116,805	118,940	117,714	120,006
CIDs with a single MNID	150,601	112,506	114,820	113,428	115,957
CIDs with multiple MNIDs	9583	4299	4120	4286	4049
With MeSH filtering					
MNIDs with CID(s)	86,895	85,122	84,212	85,058	84,098
MNIDs with a single CID	50,715	65,640	63,924	65,384	63,580
MNIDs with multiple CIDs	36,180	19,482	20,288	19,674	20,518
CIDs with MNIDs	159,958	116,733	118,871	117,645	119,940
CIDs with a single MNID	158,187	116,038	118,177	116,938	119,246
CIDs with multiple MNIDs	1771	695	694	707	694

See the text for the description of each filtering Strategy type

The use of the unfiltered synonyms for generating MNID-CID associations resulted in 9583 CIDs being associated with multiple MNIDs, which corresponds to 6% of all CIDs with MNIDs (Table 5). The synonym filtering reduced them to about four thousand CIDs, and the subsequent MeSH filtering further reduced them to around seven hundred CIDs. Note that the MeSH filtering effectively restricts that a chemical can be associated with no more than two MeSH records, because the consensus threshold for MeSH filtering was ≥ 50%.

**Fig. 7:**
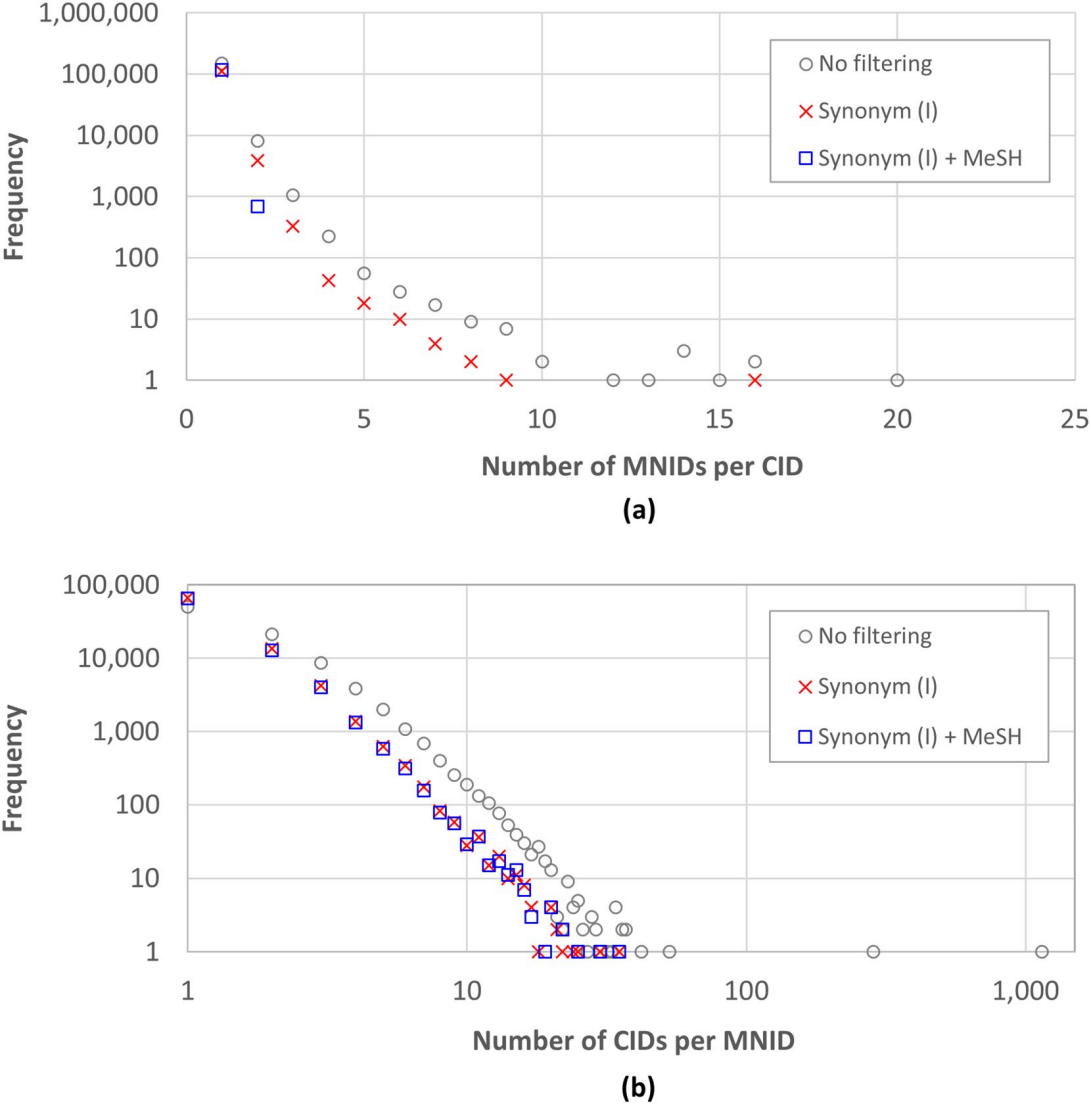
Distribution of the CID counts per MNID and the MNID count per CID: **a** The number of MeSH IDs (MNIDs) associated with a given CID and **b** the number of CIDs associated with a given MNID

**Fig. 8:**
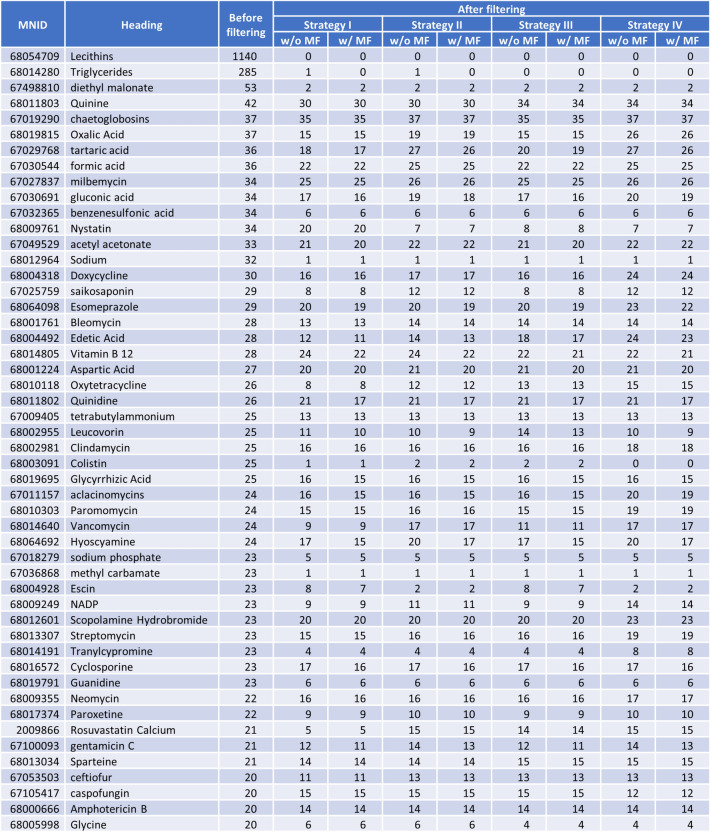
The number of CIDs associated with selected MNIDs before and after synonym and MeSH filtering. The selected MNIDs are the top-50 MNIDs with the most CIDs before filtering. The abbreviations “w/o MF” and “w/ MF” stand for “without MeSH Filtering” and “with MeSH filtering”, respectively

Figure 7 shows the distribution of the MNID counts per CID and the CID counts per MNID before and after synonym filtering. Because the four filtering strategies showed similar results in general, only the data for Strategy I are shown in Fig. 7 and those for the others are included as a supplementary material (see Additional file 1). Figure 8 lists the top-50 MeSH records associated with the most CIDs before filtering, and their fate after filtering. As shown in Fig. 7, some MeSH records were associated with hundreds or thousands of CIDs without any filtering. These extreme cases include “lecithins” (MNID 68054709) and “triglycerides” (MNID 68014280), which were associated with 1,140 CIDs and 285 CIDs, respectively (see Fig. 8). MeSH records like “lecithins” (MNID 68054709) and “triglycerides” (MNID 68014280) represent a group or class of chemicals, rather than a specific chemical, they were associated with many CIDs before filtering. These associations were removed during the filtering.

One noticeable observation from Fig. 8 is that some MeSH records representing very simple molecules (e.g., MNID 68019815 for “oxalic acid” or MNID 67030544 for “formic acid”) were associated with multiple CIDs even after the Synonym/MeSH filtering. It is primarily due to a MeSH record not representing a single term but a group of related terms. That is, in the context of this work, a MeSH record may represent a group of related chemicals. For example, MNID 68019815 (oxalic acid) has more than 40 entry terms, including “oxalic acid”, “dilithium oxalate”, “diammonium oxalate”, “chromium oxalate”, “chromium (2+) oxalate”, “chromium (3+) oxalate (3:2)”, etc. All terms are used for generating the CID-MNID associations. As a result, many MeSH records are associated with multiple CIDs even after synonym filtering and MeSH filtering. It is noteworthy that this one-to-many relationship may involve close analogues that cannot be considered the same at any of the six levels of chemical equivalency considered in this study (see Table 1). For instance, the MeSH heading “sildenafil citrate” (MNID 2009860) has entry terms like “desmethylsildenafil” and “homosildenafil”, which are similar to but distinct chemicals from “sildenafil”. Therefore, this MeSH heading is linked to the PubChem compound records corresponding to those analogues (e.g., CID 135455980 for desmethylsildenafil and CID 135565273 for homosildenafil) as well as those for sildenafil (CID 135398744) and its citrate salt (CID 135413523).

## Discussion

### Implementation of synonym filtering process in PubChem

**Fig. 9:**
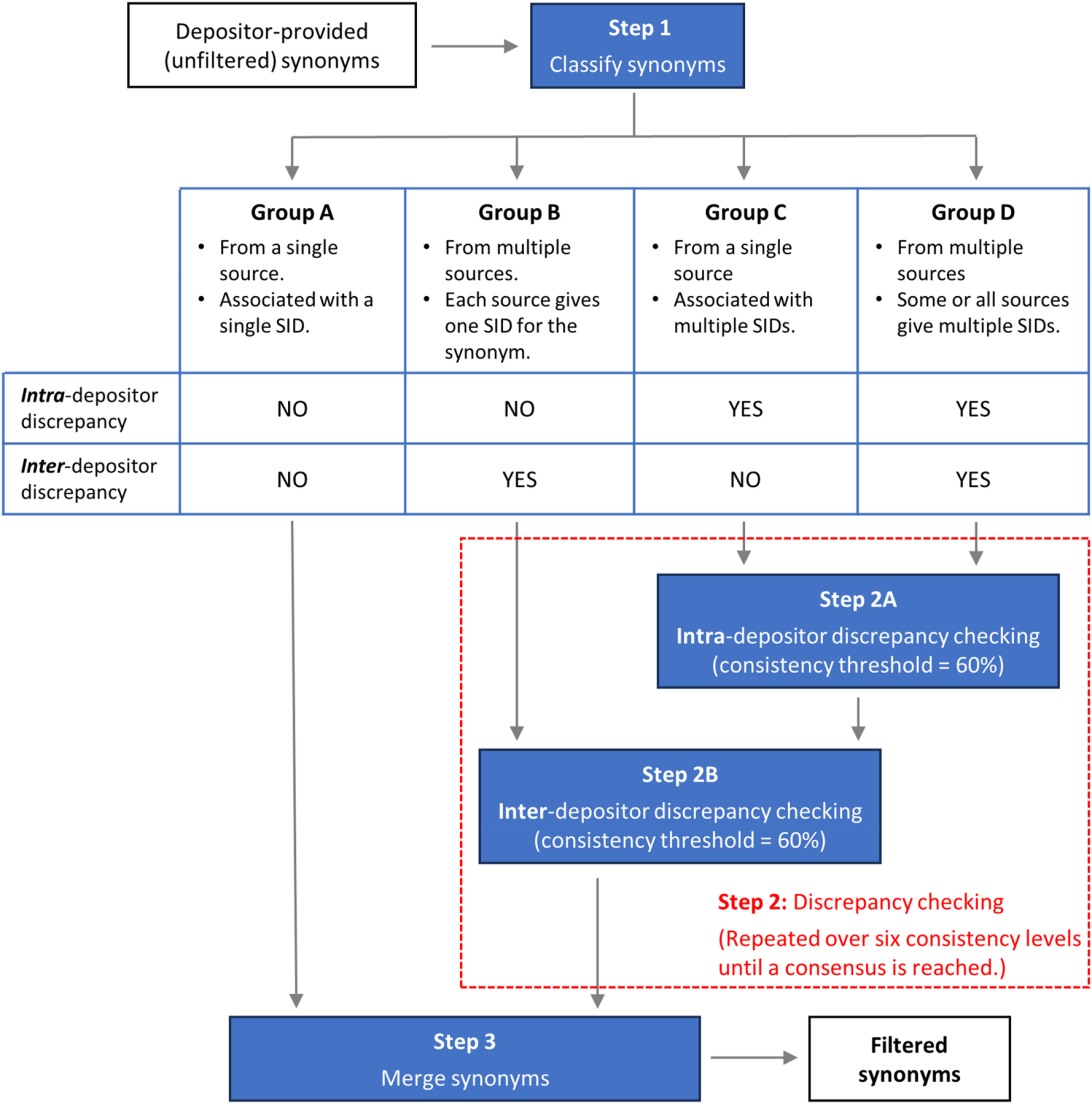
Implementation of the crowd-voting-based synonym filtering protocol in PubChem. The protocol consists of three major steps: (1) classifying synonyms into four groups, (2) resolving intra- and inter-depositor discrepancies, and (3) generating the filtered synonym set. See text for details

This study tested two consensus thresholds for crowd-voting. In practice, a threshold of 60% requires that at least two out of three depositors (or three out of four, or three out of five, …) should agree on what chemical structure a synonym means. A threshold of 70% means that at least three out of four depositors (or four out of five, or five out of six, …) should agree to reach the consensus. Our study demonstrated that the use of the lower consensus threshold makes it easier to reach the consensus, resulting in more synonyms being assigned to a single CID (see Table 3). It also disambiguated more synonym-structure associations (see Table 4). Resolving intra-depositor discrepancies before inter-depositor voting (Strategies I and II) gave better results than the multiple-vote-per-depositor approach (Strategies III and IV). These observations provide a basis for the per-synonym filtering protocol currently implemented in PubChem, which is summarized in Fig. 9. The protocol consists of the following three main steps.

1. Depositor-provided synonyms are classified into four groups, according to the number of sources that provided the synonyms and the number of SIDs associated with the synonyms.
  Group A: A synonym in this group is provided by a single depositor and associated with only one SID. The synonym has neither *intra-* nor *inter-*depositor discrepancy.
  Group B: A synonym in this group is provided by multiple depositors and each depositor provides only one SID for it. Therefore, the synonym does not have *intra*-depositor discrepancy, but has *inter*-depositor discrepancy.
  Group C: A synonym in this group is provided by a single depositor, but the depositor associates the synonym with multiple SIDs. The synonym has intra-depositor discrepancy, but no inter-depositor discrepancy.
  Group D: A synonym in this group is provided by multiple depositors and some or all sources give multiple SIDs for it. Therefore, the synonym has both intra- and inter-depositor discrepancy.
2. Synonym-structure discrepancy checking is performed against the synonyms in Groups B, C, and D. This step consists of two smaller steps: the intra-depositor discrepancy checking (step 2A in Fig. 9) against the Group C and D synonyms and the inter-depositor discrepancy checking (step 2B) against the Group B synonyms as well as those group C and D synonyms that passed through step 2A. For each synonym, step 2 is repeated over the six consistency levels (in the order of CID > STE > PCID > PSTE > CON > PCON) until a consensus is reached on the synonym-structure association. A consistency threshold of 60% is used for both the intra- and inter-depositor discrepancy resolutions. If both intra- and inter-depositor discrepancy is resolved at any chemical consistency level, the synonym is considered clean and used in the next step. The synonyms whose associated structures are inconsistent at all six consistency levels are discarded.
3. Filtered synonyms are generated by combining the Group A synonyms and those that passed through Step 2. The associations of these synonyms with their consensus structures are used to create the filtered synonym list for each compound (using the CIDs of the consensus structures).

### CID-to-MNID mapping

PubChem creates the Compound-to-MeSH mapping from filtered synonyms. When any filtered synonym of a compound matches any entry terms or registry numbers of a MeSH record, an association between the compound and MeSH records is created. Multiple CID-to-MeSH links may be generated via multiple filtered synonyms. Only the links upon which at least half of the depositors agree are selected. This MeSH filtering step restricts a compound to be associated with up to two MeSH records.

### Access to depositor-provided synonyms

**Fig. 10:**
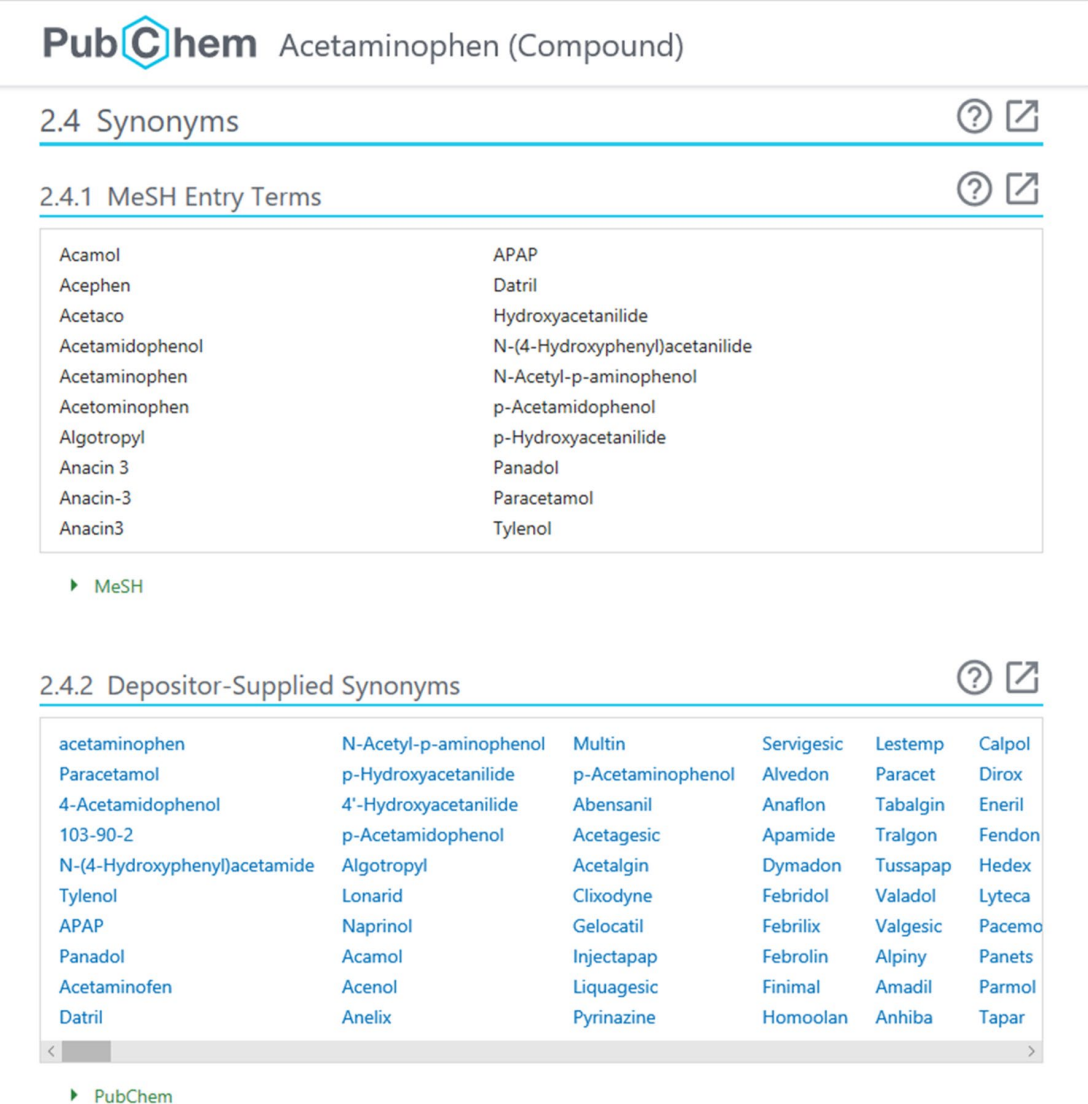
Partial screenshot of the Compound Summary page for acetaminophen (CID 1983), accessible at the: https://pubchem.ncbi.nlm.nih.gov/compound/1983#section=Synonyms. The subsection “MeSH Entry Terms” presents all entry terms for the “acetaminophen” record in MeSH (MNID 68000082). The “Depositor-Supplied Synonyms” subsection lists depositor-provided synonyms filtered using the crowd-voting strategy described in this paper

As shown in Fig. 10, the filtered synonyms for a compound can be found under the “Depositor-Supplied Synonyms” section of its Compound Summary page. When a filtered synonym of the compound matches an entry term of a MeSH record, all entry terms for that MeSH record are presented in the “MeSH entry terms” section. This section may present up to two MeSH records because of the MeSH filtering with a consensus threshold of 50%. On the other hand, the Substance Record page of a substance shows the unfiltered synonyms provided by the depositor who submitted that substance.

**Fig. 11:**
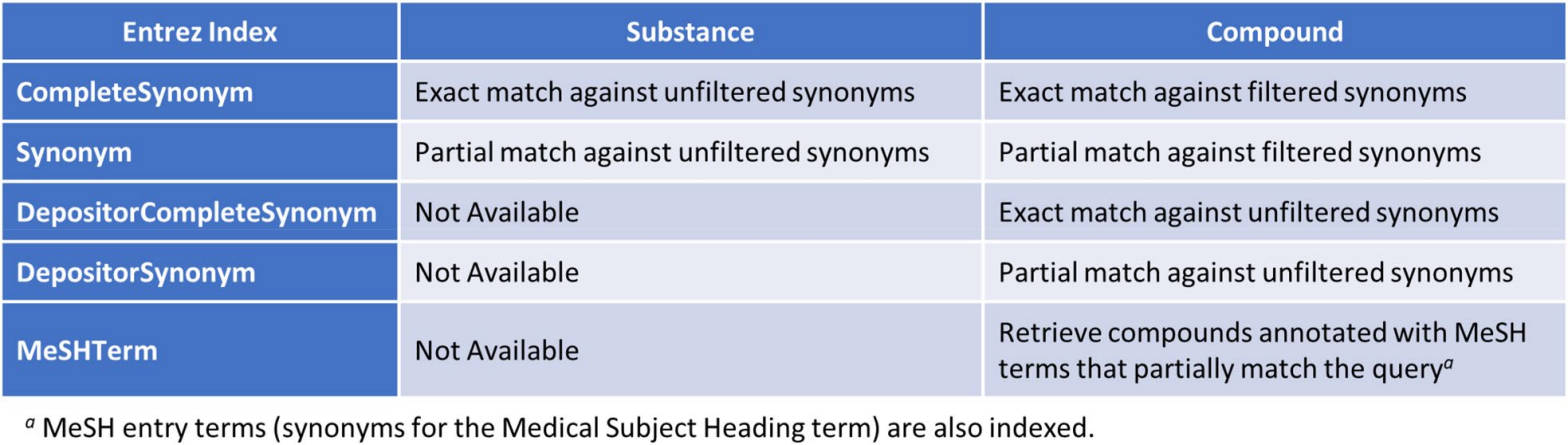
Entrez indices for searching for substance and compound records by chemical synonym or MeSH entry term

Depositor-provided synonyms are indexed for chemical search by name through the NCBI Entrez text and numeric search system [43, 44]. In Entrez, one can limit the search to an aspect (often referred to as a field) of the records, by using the Entrez index corresponding to that field. Figure 11 displays Entrez indices used for searching chemicals by name. An Entrez index is specified by suffixing the chemical name query with the name of the index enclosed with the square bracket (e.g., aspirin[synonym]). The Entrez index “Synonym” invokes a search for chemicals that contain the query string in one of its synonyms (that is, partial matching). The Entrez Index “CompleteSynonym” is used to search for chemicals whose synonym exactly matches the query chemical name. It should be noted that, both Entrez indices “Synonym” and “CompleteSynonym” invoke a search against *filtered* synonyms in the Compound database, while these indices look up *unfiltered* synonyms in the Substance database. To search against unfiltered synonyms in the Compound database, Entrez indices “DepositorSynonym” or “DepositorCompleteSynonym” should be used. An Entrez Index “MeSHTerm” is used to search the Compound database for chemicals annotated with a MeSH term that partially (or fully) matches the query.

The PubChem Identifier Exchange Service (https://pubchem.ncbi.nlm.nih.gov/idexchange/) [10] converts one type of identifier for a given set of chemical structures into a different type of identifier for identical or similar chemical structures. This tool can be used to convert a set of chemical synonyms into their chemical structures (represented by CIDs, SMILES or InChI strings). In the initial step, the input chemical synonyms are mapped with the *filtered* synonyms of the existing compounds in PubChem. Then, the mapped compounds are subject to the requested operation (e.g., getting identical or related compounds) and the resulting records are returned in a specified format (e.g., CIDs, SMILES, InChI). In essence, the Identifier Exchange Service allows the user to readily perform a batch of chemical name searches. More detailed explanation of this tool can be found in our recent paper [10].

Depositor-provided synonyms may be retrieved programmatically through PUG-REST [45, 46], which is a Representational State Transfer (REST)-like interface [47, 48]. It also supports search by chemical name. Note that filtered synonyms are retrieved for compounds or looked up during search against the Compound database through PUG-REST. In contrast, it is unfiltered synonyms that are retrieved or looked up when the user deals with records in the Substance database. The MeSH entry terms associated with a CID (the upper portion of Fig. 10) can be downloaded through PUG-View [49], which is another REST-like interface specialized in accessing the annotations for a PubChem record.

In addition, all PubChem’s unfiltered and filtered synonyms are freely available for bulk download within the PubChem FTP site (these files are available at this location: https://ftp.ncbi.nlm.nih.gov/pubchem/Compound/Extras/).

### Limitation of synonym filtering

PubChem’s synonym filtering process is designed to check the consistency of chemical synonym-structure associations provided by depositors, but not the accuracy of them. Checking the accuracy of synonym-structure associations inevitably requires substantial manual curation efforts in addition to cross-comparison to curated and authoritative data sources. However, manual curation is not a feasible option because PubChem contains many millions of compounds. In addition, many compounds contain hundreds of synonyms provided by hundreds of depositors and are not found in any curated and/or authoritative data sources. With that said, the synonym filtering process is a critical step in data quality control of PubChem name-structure relationships.

It is worth noting that the methods described here can be easily defeated when PubChem data contributors (blindly) copy from each other (which unfortunately happens too often). If an error is copied and then contributed to PubChem multiple times, correct information can be overwhelmed by the crowd-voting scheme from contributors with faulty information. This has led PubChem to implement so-called, white-lists and black-lists for name-structure associations. When an error is reported to PubChem [for example, through the NLM Support Center (https://support.nlm.nih.gov)] and the correct information is known, a white-list entry is made to ensure a name-structure association cannot be changed by erroneous data contributors. If the correct information is not known (or cannot be mapped to a chemical structure in PubChem), a black-list entry is made to prevent a name-structure association from ever occurring.

It is not hard to imagine various improvements that can be made to PubChem crowd-voting-based approaches. One key method researched by the PubChem team is to group chemical names together that refer to the same ‘chemical concept’. In this context, a chemical concept is nothing more than a group of chemical names that refer to the same chemical substance. One can then apply the methodology described in this paper to assign a single chemical structure to a group of chemical names. A problem with this approach is the use of a single chemical name to refer to multiple chemical substances. In addition, there are often multiple (legitimate) chemical structure representations for the same chemical substance. Lastly, such a concept-based approach is very sensitive to the highly varied use cases of chemical names and their corresponding structural descriptions to varying degrees of exactness, such as referring to a drug and its salt form in the same manner. While a complete description of this "concept" approach is beyond the scope of this work and is still a work in progress within PubChem, it builds upon the lessons learned and the general applicability to this “crowdsourcing” approach of improving the quality of name-structure relationships in PubChem.

### Filtering non-chemical names

Sometimes synonyms provided by depositors are not necessarily chemical names. Several types of these names are often observed, including protein/gene names, antibody names, disease names, plant names, and organism names. These names are provided together with chemical structures because they are related to the chemicals to some extent. For example, garlic and ginseng are plant names and they are commonly assigned to the natural products (chemicals) extracted from them. For such cases, specific vocabularies, such as known gene/protein/enzyme names (130 thousand from UniProt [50]) and organism names (2.5 million from NCBI Taxonomy [51]), are harnessed to filter them out. Additionally, there are chemical class names that are for a certain type of chemicals, rather than for specific chemical substances, *e.g.*, “indoles” or “ketones”, which are also filtered out. Sometimes a name can legitimately have multi-meanings in various scientific fields or subdisciplines. This is especially true for acronyms and chemical names with five characters or less (e.g., “DNA” for “dinitro-aniline”, thus any chemical name filtering procedure should be used with great caution.

### PubChem Search with a chemical name query

PubChem Search, available at the PubChem homepage (https://pubchem.ncbi.nlm.nih.gov), is a unified search interface that allows users to perform a keyword search against PubChem’s multiple data collections (e.g., Compound, Substance, BioAssay, Gene, Protein, Pathway, Cell Line, Taxonomy, and Patent). When a keyword query is provided, PubChem Search goes through the indexed fields of each data collection to identify records containing the query string and presents matched records from each collection. The search result page has tabs that allow the user to view the matched records from different collections. In addition, when possible, PubChem Search tries to identify the most relevant record and display it at the top of the search result page. More detailed explanation on PubChem Search can be found in our previous paper [9].

It is worth mentioning that the backend databases used by PubChem Search are different from those used by the PubChem Entrez interface (see the “Access to depositor-provided synonyms” section). In PubChem Search, while the unfiltered depositor-provided synonyms are indexed for the Substance collection, the filtered synonyms are indexed for the Compound collection. Other types of chemical names and identifiers, collected from authoritative sources [such as the U.S. Food and Drug Administration (FDA)’s UNique Ingredient Identifier (UNII) or the European Community (EC) Number] or generated with third-party software (e.g., systematic IUPAC names or InChI/InChIKey strings), are also indexed for search of the Compound collection in PubChem Search. Importantly, to overcome the limitation of synonym filtering, PubChem Search adopts multiple approaches, such as the use of blacklists, whitelists, and chemical concepts, which are not used in the Entrez search system. Therefore, the results from PubChem Search and Entrez are not always the same.

## Conclusion

Mapping from synonyms to chemical structures in PubChem relies upon synonym-structure associations provided by individual depositors. However, substantial discrepancies exist in these associations within and between depositors, resulting in concerns by researchers over data quality in PubChem. The present paper describes the basis for the PubChem synonym filtering process and its application for CID-MNID mapping with MeSH.

The PubChem chemical synonym filtering process was developed based on the analysis of four different crowd-voting strategies (Table 2) that utilize the consistency of chemical structure associations. The four crowd-voting strategies differ in how to deal with intra-depositor discrepancies (a single vote per depositor vs. multiple votes per depositor) and the consistency threshold value used (60% vs 70%). The agreement of “voting” was defined at six levels, in the order of specificity: same exact structure (CID), same stereo form (STE), same exact parent structure (PCID), same parent stereo (PSTE), same connectivity (CON), and same parent connectivity (PCON) (Table 1). While all four strategies showed similar results, Strategy I (one vote per depositor with a 60% consistency threshold) resulted in the largest number of synonyms that can be assigned to a single CID as well as the greatest number of synonym-structure associations disambiguated at the six chemical equivalency contexts. Strategy I is employed in the current PubChem synonym filtering that is in use today.

The filtered synonyms for each *compound* can be found under the “Depositor-Supplied Synonyms” section of its Compound Summary page. When available, the synonyms that match MeSH terms are also displayed under the “MeSH Entry Terms” section. On the other hand, the “Depositor-Supplied Synonyms” section of the Substance Record page of a substance shows all unfiltered synonyms provided for that substance record by the depositor. Using Entrez indices listed in Fig. 11, one can search the Compound database by either filtered or unfiltered synonym and the Substance database by unfiltered synonym.

The PubChem synonym filtering process is designed to look for consensus in name-structure associations, but not for its correctness. As a result, it can fail to recognize incorrect chemical name-structure associations. However, this filtering process is an important starting point for quality control in name-structure associations in large chemical databases like PubChem that have many data sources.

### Supplementary Information

Additional file 1.

## Data Availability

For reproducibility, the unfiltered SID-synonym association data set used in this work is made available at Zenodo (10.5281/zenodo.11194943) [52]. PubChem’s up-to-date unfiltered and filtered synonyms are freely available for bulk download via the PubChem FTP site (https://ftp.ncbi.nlm.nih.gov/pubchem/Substance/Extras/ and https://ftp.ncbi.nlm.nih.gov/pubchem/Compound/Extras/).

## References

[1] Kim S, Chen J, Cheng T, Gindulyte A, He J, He S, Li Q, Shoemaker BA, Thiessen PA, Yu B, Zaslavsky L, Zhang J, Bolton EE (2023). PubChem 2023 update. Nucleic Acids Res.

[2] Kim S (2016). Getting the most out of PubChem for virtual screening. Expert Opin Drug Discov.

[3] Sayers Eric W, Beck J, Bolton Evan E, Brister JR, Chan J, Comeau Donald C, Connor R, DiCuccio M, Farrell Catherine M, Feldgarden M, Fine Anna M, Funk K, Hatcher E, Hoeppner M, Kane M, Kannan S, Katz Kenneth S, Kelly C, Klimke W, Kim S, Kimchi A, Landrum M, Lathrop S, Lu Z, Malheiro A, Marchler-Bauer A, Murphy Terence D, Phan L, Prasad Arjun B, Pujar S, Sawyer A, Schmieder E, Schneider Valerie A, Schoch Conrad L, Sharma S, Thibaud-Nissen F, Trawick Barton W, Venkatapathi T, Wang J, Pruitt Kim D, Sherry Stephen T (2024). Database resources of the National Center for Biotechnology Information. Nucleic Acids Res.

[4] Kim S, Thiessen PA, Bolton EE, Chen J, Fu G, Gindulyte A, Han L, He J, He S, Shoemaker BA, Wang J, Yu B, Zhang J, Bryant SH (2016). PubChem substance and compound databases. Nucleic Acids Res.

[5] Wang YL, Bryant SH, Cheng TJ, Wang JY, Gindulyte A, Shoemaker BA, Thiessen PA, He SQ, Zhang J (2017). PubChem BioAssay: 2017 update. Nucleic Acids Res.

[6] Kim S, Cheng T, He S, Thiessen PA, Li Q, Gindulyte A, Bolton EE (2022). PubChem protein, gene, pathway, and taxonomy data collections: bridging biology and chemistry through target-centric views of PubChem data. J Mol Biol.

[7] Kim S, Chen J, Cheng T, Gindulyte A, He J, He S, Li Q, Shoemaker BA, Thiessen PA, Yu B, Zaslavsky L, Zhang J, Bolton EE (2021). PubChem in 2021: new data content and improved web interfaces. Nucleic Acids Res.

[8] Kim S, Chen J, Cheng TJ, Gindulyte A, He J, He SQ, Li QL, Shoemaker BA, Thiessen PA, Yu B, Zaslavsky L, Zhang J, Bolton EE (2019). PubChem 2019 update: improved access to chemical data. Nucleic Acids Res.

[9] Kim S (2021). Exploring chemical information in PubChem. Curr Prot.

[10] Kim S, Bolton EE, Daina A, Przewosny M, Zoete V (2023). PubChem: a large-scale public chemical database for drug discovery. Open access databases and datasets for drug discovery, methods and principles in medicinal chemistry.

[11] Medical Subject Headings. https://www.ncbi.nlm.nih.gov/mesh. Accessed 3 Jun 2020.

[12] Kim S, Thiessen PA, Cheng T, Yu B, Shoemaker BA, Wang JY, Bolton EE, Wang YL, Bryant SH (2016). Literature information in PubChem: associations between PubChem records and scientific articles. J Cheminform.

[13] Akhondi SA, Kors JA, Muresan S (2012). Consistency of systematic chemical identifiers within and between small-molecule databases. J Cheminform.

[14] Williams AJ, Ekins S (2011). A quality alert and call for improved curation of public chemistry databases. Drug Discov Today.

[15] Fourches D, Muratov E, Tropsha A (2010). Trust, but verify: on the importance of chemical structure curation in cheminformatics and QSAR modeling research. J Chem Inf Model.

[16] Williams AJ, Ekins S, Tkachenko V (2012). Towards a gold standard: regarding quality in public domain chemistry databases and approaches to improving the situation. Drug Discov Today.

[17] Kramer C, Lewis R (2012). QSARs, data and error in the modern age of drug discovery. Curr Top Med Chem.

[18] The rise of crowdsourcing http://www.wired.com/wired/archive/14.06/crowds.html.

[19] Delbourgo J. Listing people. Isis; an international review devoted to the history of science and its cultural influences.2012; 103(4):735–742. 10.1086/669046 23488241

[20] Dekkers OM, Mummery CL, Rabelink TJ (2014). A case for crowd sourcing in stem cell research. Stem Cells Transl Med.

[21] Prill RJ, Saez-Rodriguez J, Alexopoulos LG, Sorger PK, Stolovitzky G (2011). Crowdsourcing network inference: the DREAM predictive signaling network challenge. Sci Signal.

[22] Berens P, Freeman J, Deneux T, Chenkov N, McColgan T, Speiser A, Macke JH, Turaga SC, Mineault P, Rupprecht P, Gerhard S, Friedrich RW, Friedrich J, Paninski L, Pachitariu M, Harris KD, Bolte B, Machado TA, Ringach D, Stone J, Rogerson LE, Sofroniew NJ, Reimer J, Froudarakis E, Euler T, Roson MR, Theis L, Tolias AS, Bethge M (2018). Community-based benchmarking improves spike rate inference from two-photon calcium imaging data. PLoS Comput Biol.

[23] Kuhlmann L, Karoly P, Freestone DR, Brinkmann BH, Temko A, Barachant A, Li F, Titericz G, Lang BW, Lavery D, Roman K, Broadhead D, Dobson S, Jones G, Tang QN, Ivanenko I, Panichev O, Proix T, Nahlik M, Grunberg DB, Reuben C, Worrell G, Litt B, Liley DTJ, Grayden DB, Cook MJ (2018). Epilepsyecosystem.org: crowd-sourcing reproducible seizure prediction with long-term human intracranial EEG. Brain.

[24] Elgin SCR, Hauser C, Holzen TM, Jones C, Kleinschmit A, Leatherman J, Genomics Educ P (2017). The GEP: crowd-sourcing big data analysis with undergraduates. Trends Genet.

[25] Vezzi F, Narzisi G, Mishra B (2012). Reevaluating assembly evaluations with feature response curves: GAGE and assemblathons. PLoS ONE.

[26] Martin SF, Falkenberg H, Dyrlund TF, Khoudoli GA, Mageean CJ, Linding R (2013). PROTEINCHALLENGE: crowd sourcing in proteomics analysis and software development. J Proteomics.

[27] Vashisht R, Mondal AK, Jain A, Shah A, Vishnoi P, Priyadarshini P, Bhattacharyya K, Rohira H, Bhat AG, Passi A, Mukherjee K, Choudhary KS, Kumar V, Arora A, Munusamy P, Subramanian A, Venkatachalam A, Gayathri S, Raj S, Chitra V, Verma K, Zaheer S, Balaganesh J, Gurusamy M, Razeeth M, Raja I, Thandapani M, Mevada V, Soni R, Rana S, Ramanna GM, Raghavan S, Subramanya SN, Kholia T, Patel R, Bhavnani V, Chiranjeevi L, Sengupta S, Singh PK, Atray N, Gandhi S, Avasthi TS, Nisthar S, Anurag M, Sharma P, Hasija Y, Dash D, Sharma A, Scaria V, Thomas Z, Chandra N, Brahmachari SK, Bhardwaj A (2012). Crowd sourcing a new paradigm for interactome driven drug target identification in *Mycobacterium tuberculosis*. PLoS ONE.

[28] Khare R, Good BM, Leaman R, Su AI, Lu ZY (2016). Crowdsourcing in biomedicine: challenges and opportunities. Brief Bioinform.

[29] McCoy AB, Wright A, Rogith D, Fathiamini S, Ottenbacher AJ, Sittig DF (2014). Development of a clinician reputation metric to identify appropriate problem-medication pairs in a crowdsourced knowledge base. J Biomed Inform.

[30] Conway KR, Boddy CN (2013). ClusterMine360: a database of microbial PKS/NRPS biosynthesis. Nucleic Acids Res.

[31] Luengo-Oroz MA, Arranz A, Frean J (2012). Crowdsourcing malaria parasite quantification: an online game for analyzing images of infected thick blood smears. J Med Internet Res.

[32] Kaminuma E, Baba Y, Mochizuki M, Matsumoto H, Ozaki H, Okayama T, Kato T, Oki S, Fujisawa T, Nakamura Y, Arita M, Ogasawara O, Kashima H, Takagi T (2020). DDBJ Data Analysis Challenge: a machine learning competition to predict Arabidopsis chromatin feature annotations from DNA sequences. Genes Genet Syst.

[33] Picache JA, May JC, McLean JA (2020). Crowd-sourced chemistry: considerations for building a standardized database to improve omic analyses. ACS Omega.

[34] Esteban O, Blair RW, Nielson DM, Varada JC, Marrett S, Thomas AG, Poldrack RA, Gorgolewski KJ (2019). Crowdsourced MRI quality metrics and expert quality annotations for training of humans and machines. Sci Data.

[35] Karp PD (2016). Crowd-sourcing and author submission as alternatives to professional curation. Database.

[36] Lesurf R, Cotto KC, Wang G, Griffith M, Kasaian K, Jones SJM, Montgomery SB, Griffith OL, A Open Regulatory (2016). ORegAnno 30: a community-driven resource for curated regulatory annotation. Nucleic Acids Res.

[37] Tastan O, Qi YJ, Carbonell JG, Klein-Seetharaman J, Altman RB, Dunker AK, Hunter L, Ritchie MD, Murray T, Klein TE (2015). Refining literature curated protein interactions using expert opinions. Pacific Symposium on Biocomputing 2015. Biocomputing-Pacific Symposium on Biocomputing.

[38] Waldispuhl J, Kam A, Gardner PP, Altman RB, Dunker AK, Hunter L, Ritchie MD, Murray T, Klein TE (2015). Crowdsourcing rna structural alignments with an online computer game. Pacific Symposium on Biocomputing 2015. Biocomputing-Pacific Symposium on Biocomputing.

[39] Burger JD, Doughty E, Khare R, Wei CH, Mishra R, Aberdeen J, Tresner-Kirsch D, Wellner B, Kann MG, Lu Z, Hirschman L (2014). Hybrid curation of gene-mutation relations combining automated extraction and crowdsourcing. Database.

[40] Hähnke VD, Kim S, Bolton EE (2018). PubChem chemical structure standardization. J Cheminform.

[41] Ihlenfeldt WD, Gasteiger J (1994). Hash codes for the identification and classification of molecular-structure elements. J Comput Chem.

[42] Ihlenfeldt WD, Takahashi Y, Abe H, Sasaki S (1994). Computation and management of chemical-properties in cactvs—an extensible networked approach toward modularity and compatibility. J Chem Inf Comput Sci.

[43] Schuler GD, Epstein JA, Ohkawa H, Kans JA (1996). Entrez: molecular biology database and retrieval system. Methods Enzymol.

[44] McEntyre J (1998). Linking up with Entrez. Trends Genet TIG.

[45] Kim S, Thiessen PA, Bolton EE, Bryant SH (2015). PUG-SOAP and PUG-REST: web services for programmatic access to chemical information in PubChem. Nucleic Acids Res.

[46] Kim S, Thiessen PA, Cheng T, Yu B, Bolton EE (2018). An update on PUG-REST: RESTful interface for programmatic access to PubChem. Nucleic Acids Res.

[47] Fielding RT, Taylor RN (2000) Principled design of the modern Web architecture. In: Proceedings of the 22nd International Conference on Software engineering. pp. 407–416. 10.1145/337180.337228

[48] Fielding RT (2000) Representational state transfer (REST). In: Architectural styles and the design of network-based software architectures. University of California, Irvine

[49] Kim S, Thiessen PA, Cheng TJ, Zhang J, Gindulyte A, Bolton EE (2019). PUG-view: programmatic access to chemical annotations integrated in PubChem. J Cheminform.

[50] The UniProt Consortium (2023). UniProt: the universal protein knowledgebase in 2023. Nucleic Acids Res.

[51] Schoch CL, Ciufo S, Domrachev M, Hotton CL, Kannan S, Khovanskaya R, Leipe D, Mcveigh R, O’Neill K, Robbertse B, Sharma S, Soussov V, Sullivan JP, Sun L, Turner S, Karsch-Mizrachi I (2020). NCBI Taxonomy: a comprehensive update on curation, resources and tools. Database.

[52] Kim S, Yu B, Li Q, Bolton EE (2024). Zenodo.

