# Supplementary material for: PubChem synonym filtering process using crowdsourcing
Source: J Cheminform. 2024 Jun 16;16:69. doi: 10.1186/s13321-024-00868-3 (PMC11181558; doi:10.1186/s13321-024-00868-3)
Supplement: Supplementary file 1 — Additional file 1. [file 13321_2024_868_MOESM1_ESM.docx]

# PubChem Synonym Filtering Process using Crowdsourcing

Sunghwan Kim, Bo Yu, Qingliang Li and Evan E. Bolton

National Center for Biotechnology Information, National Library of Medicine, National Institutes of Health, Bethesda, MD 20894, USA

**Supplementary Materials**

**Figure S1**. Distribution of the number of SIDs per synonym before and after synonym filtering using Strategies I through IV.

**Figure S2**. Distribution of the number of CIDs per synonym before and after synonym filtering using Strategies I through IV.

**Figure S3**. Frequency of the number of MNIDs per CID before and after synonym and MeSH filtering using Strategies I through IV.

**Figure S4**. Frequency of the number of CIDs per MNID before and after synonym and MeSH filtering using Strategies I through IV.


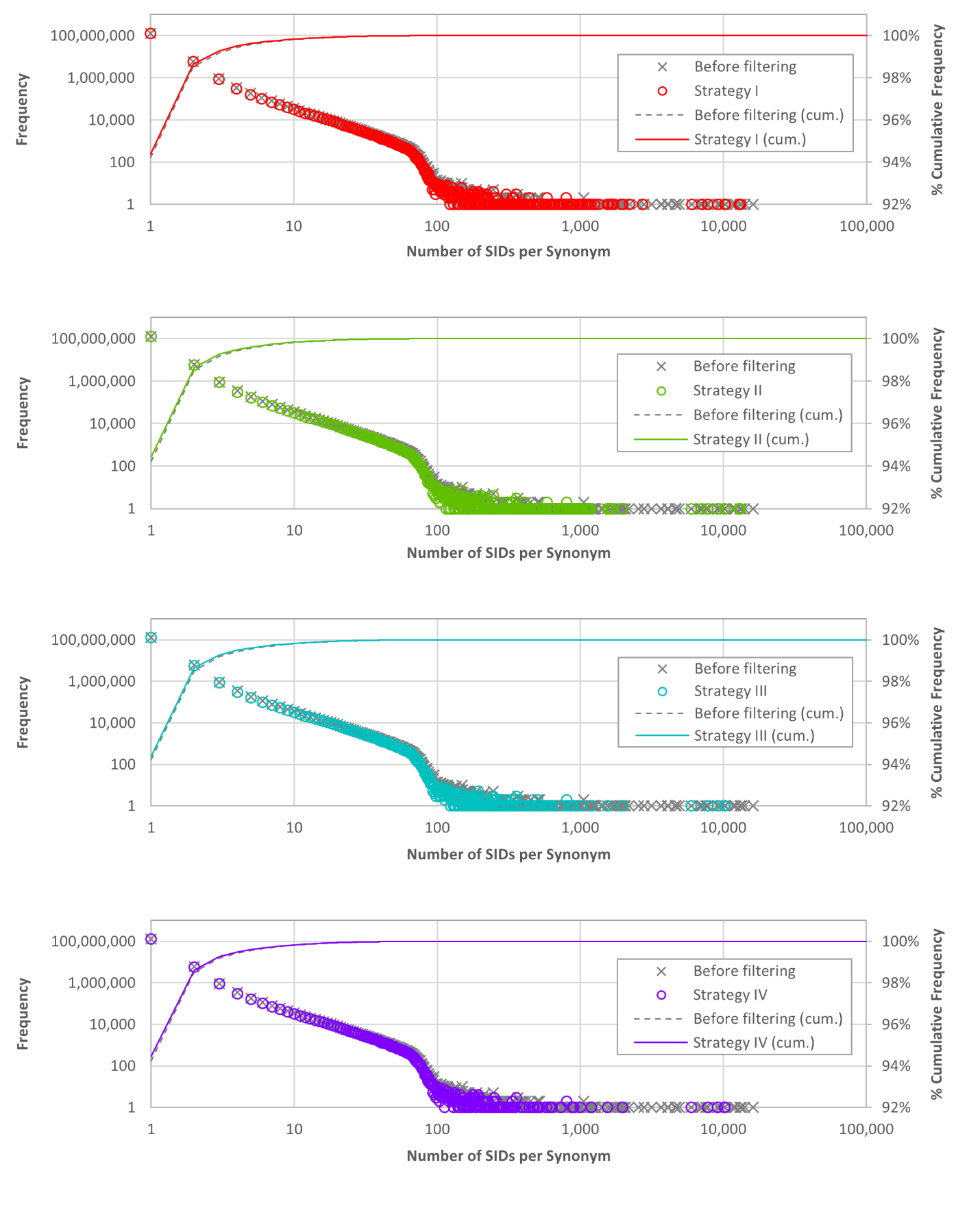


**Figure S1**. Distribution of the number of SIDs per synonym before and after synonym filtering using Strategies I through IV.


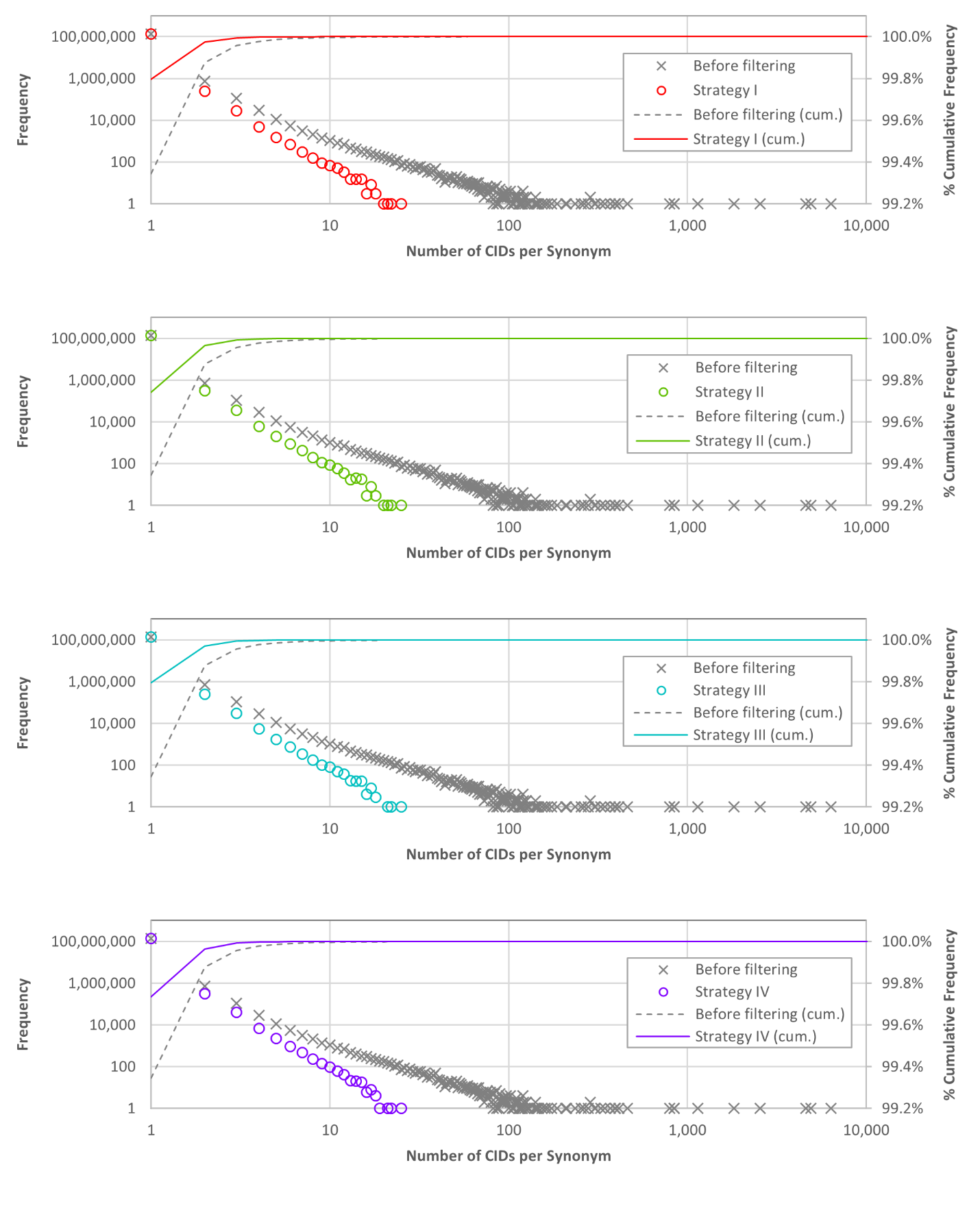


**Figure S2**. Distribution of the number of SIDs per synonym before and after synonym filtering using Strategies I through IV.


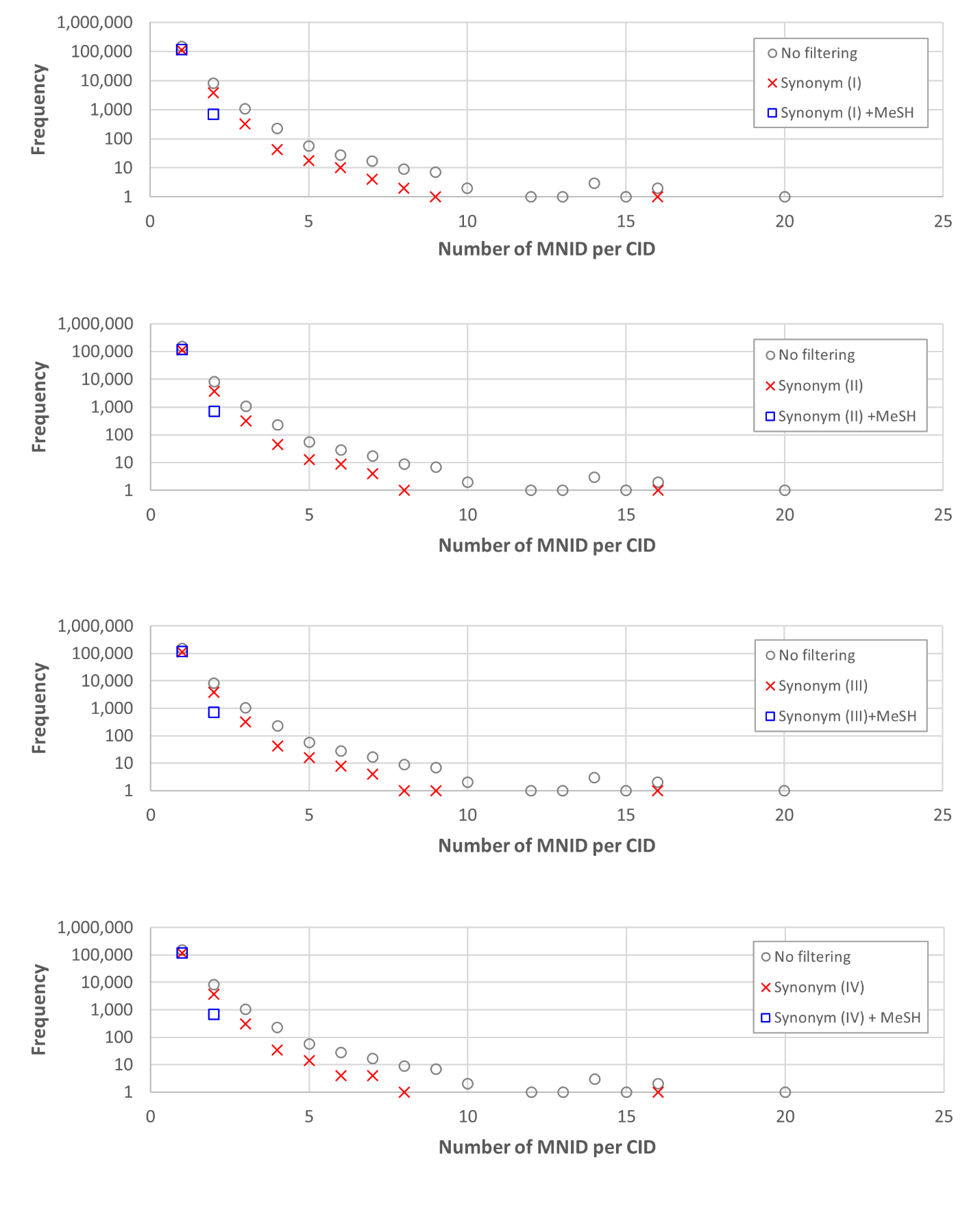


**Figure S3**. Frequency of the number of MNIDs per CID before and after synonym and MeSH filtering using Strategies I through IV.


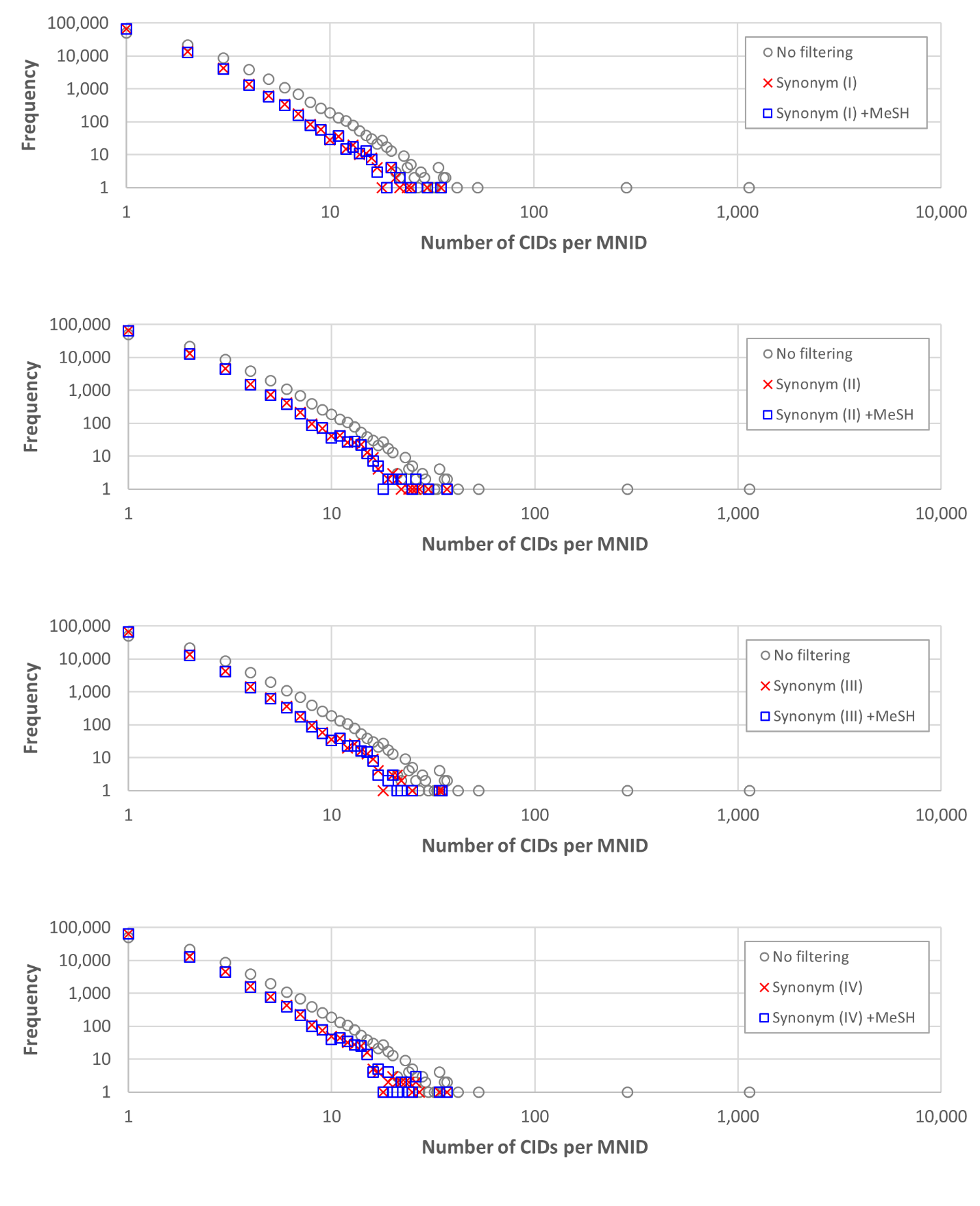


**Figure S4**. Frequency of the number of CIDs per MNID before and after synonym and MeSH filtering using Strategies I through IV.
